# Supplementary figures and images for: Abiotic stress destabilizes the bacterial community of sugar kelp, Saccharina latissima (Phaeophyceae)
Source: J Phycol. 2025 May 28;61(4):840–57. doi: 10.1111/jpy.70033 (PMC12351371; doi:10.1111/jpy.70033)

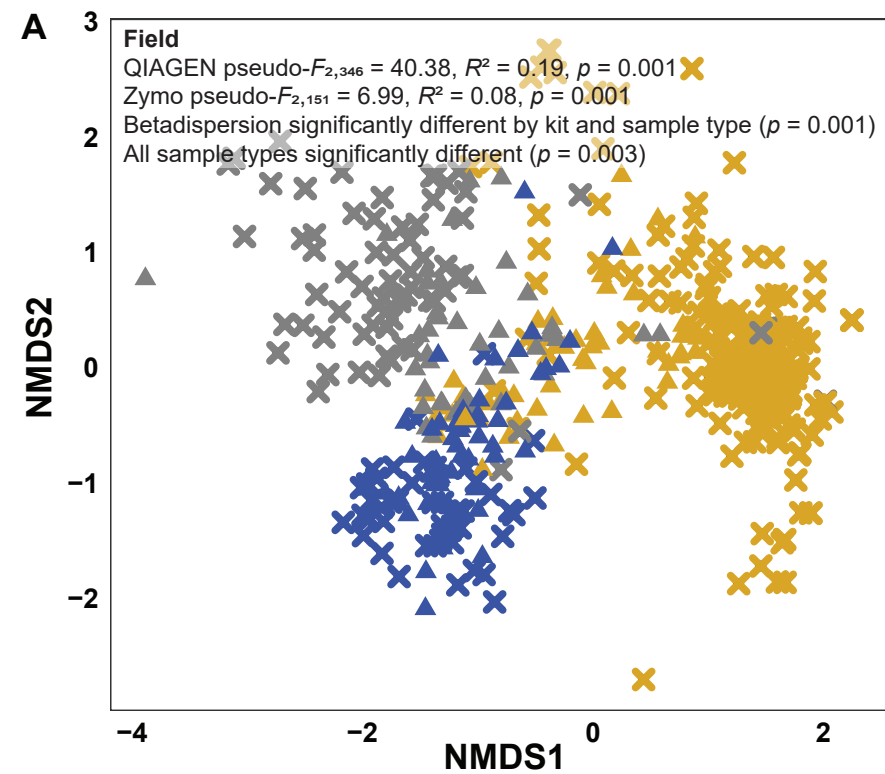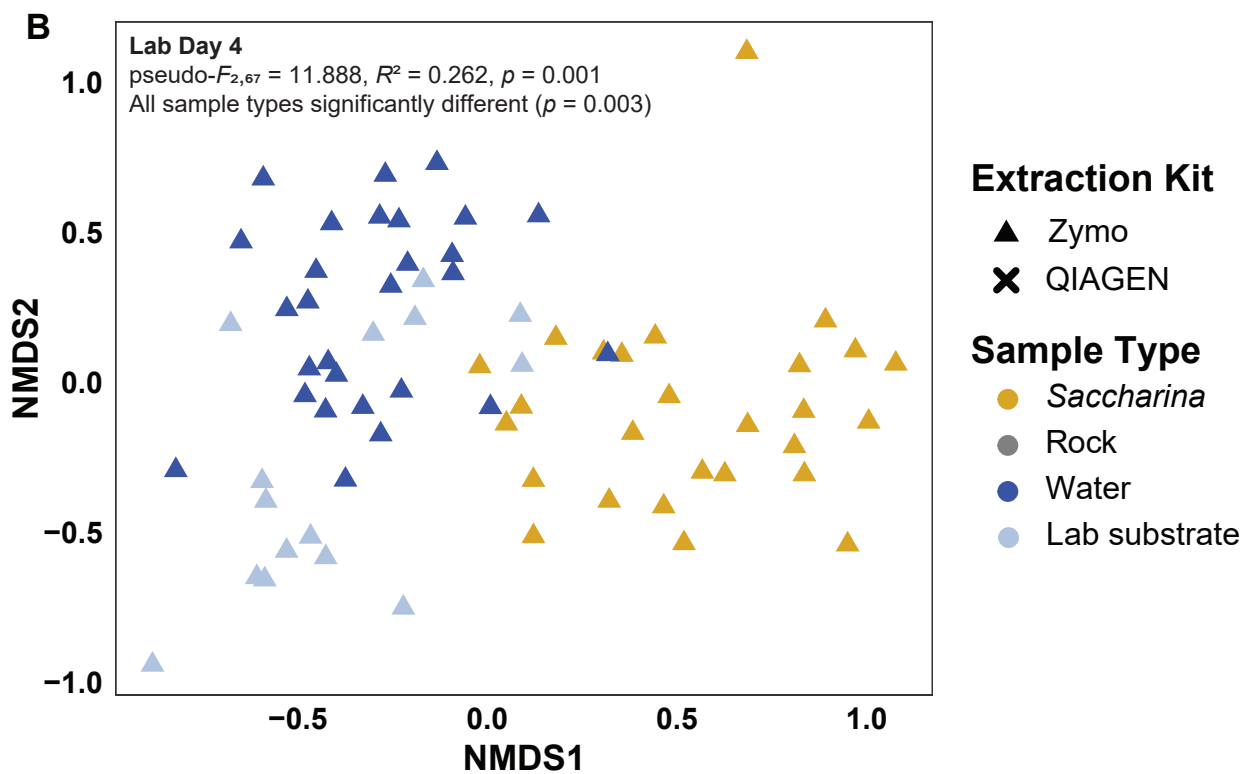

Supplement: Supplementary file 1 — Figure S1. NMDS plots showing differences by sample type (color) and the extraction kit (shape) for (A) both years of field samples and (B) lab Day 4 samples. PERMANOVA outputs are in the corresponding panels. [file JPY-61-840-s002.pdf]

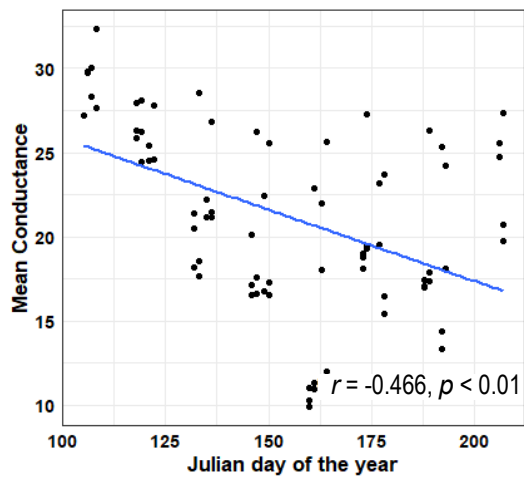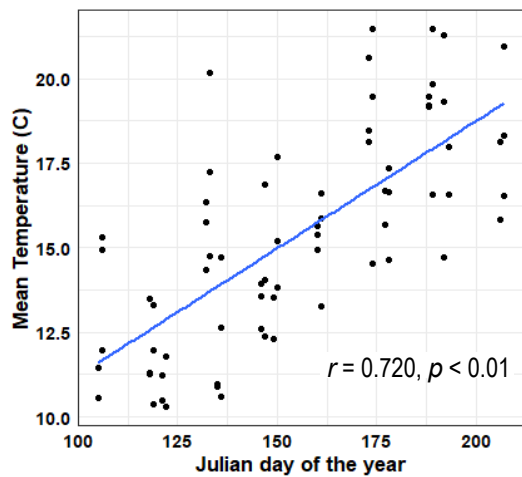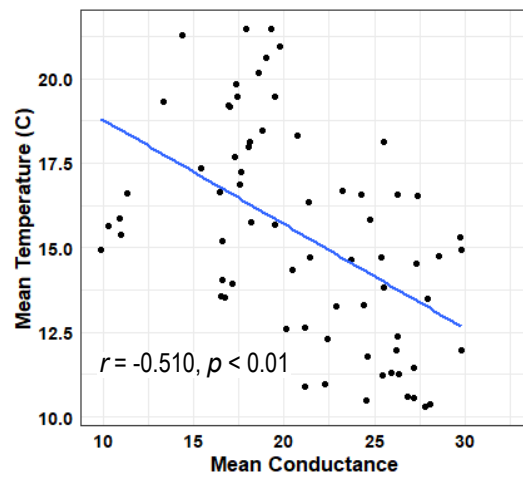

Supplement: Supplementary file 2 — Figure S2. Scatterplots with regression line showing the correlation between conductance (μS/cm; referred to as salinity in the text), temperature (°C), and Julian day for both 2021 and 2022. Results of Pearson's correlation test indicated in the plot area. [file JPY-61-840-s005.pdf]

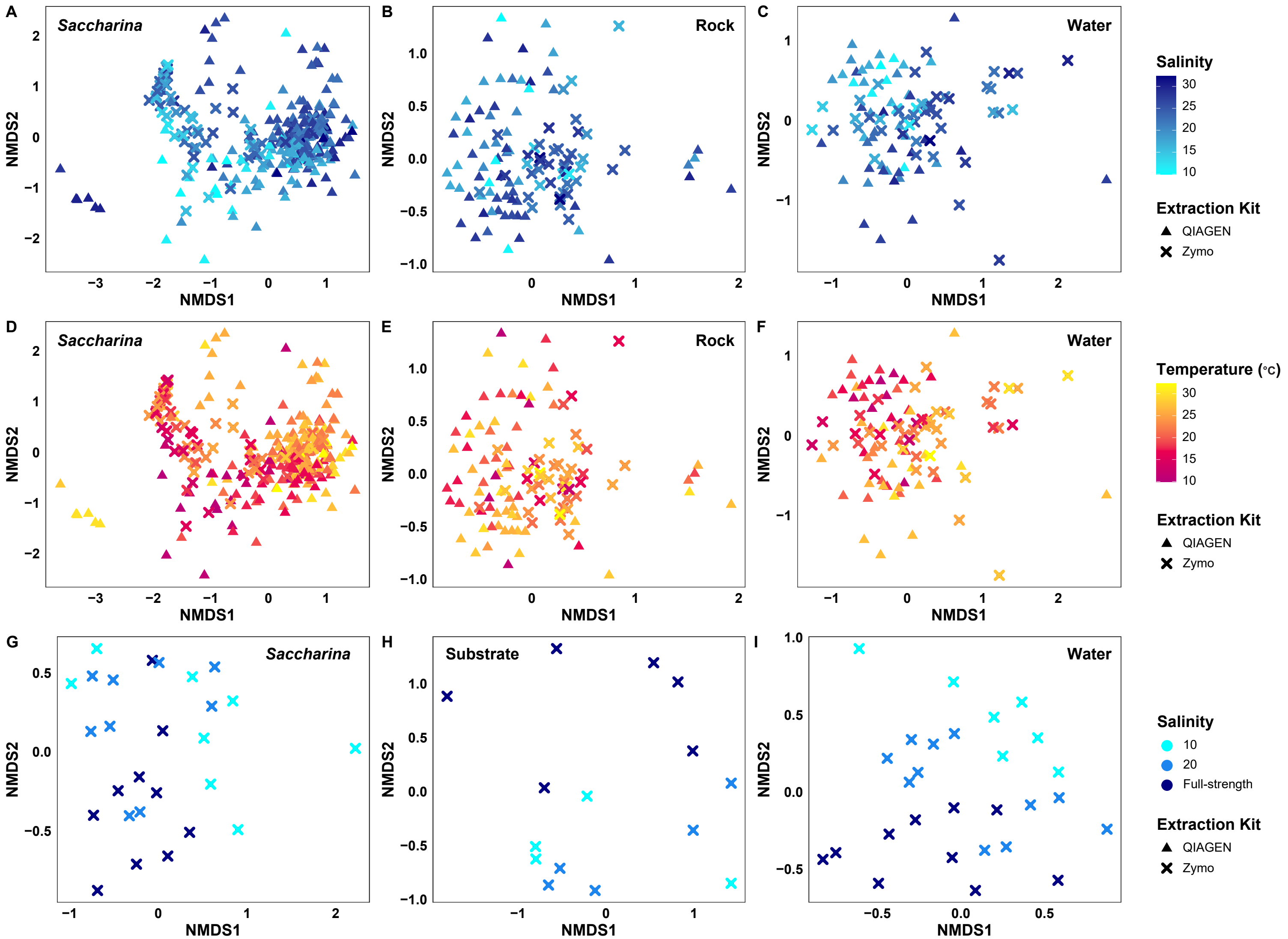

Supplement: Supplementary file 3 — Figure S3. NMDS plots by sample type showing the distribution of samples across the salinity (A:C, G:H) and temperature (D:F) gradients in the study. Panels A:C show field samples colored by salinity gradient and D:F show the same plots colored by temperature gradient. Panels G:I show lab Day 4 samples colored by salinity treatment. Note that sample number in the lab experiment are lower than the expected 16 per treatment group per sample type because of sequencing failures (sample numbers in Table S1). Point shapes indicate the extraction kit used. Corresponding output of PERMANOVA for the field (Table 1) and lab (Table 2) are in the main text. [file JPY-61-840-s001.pdf]

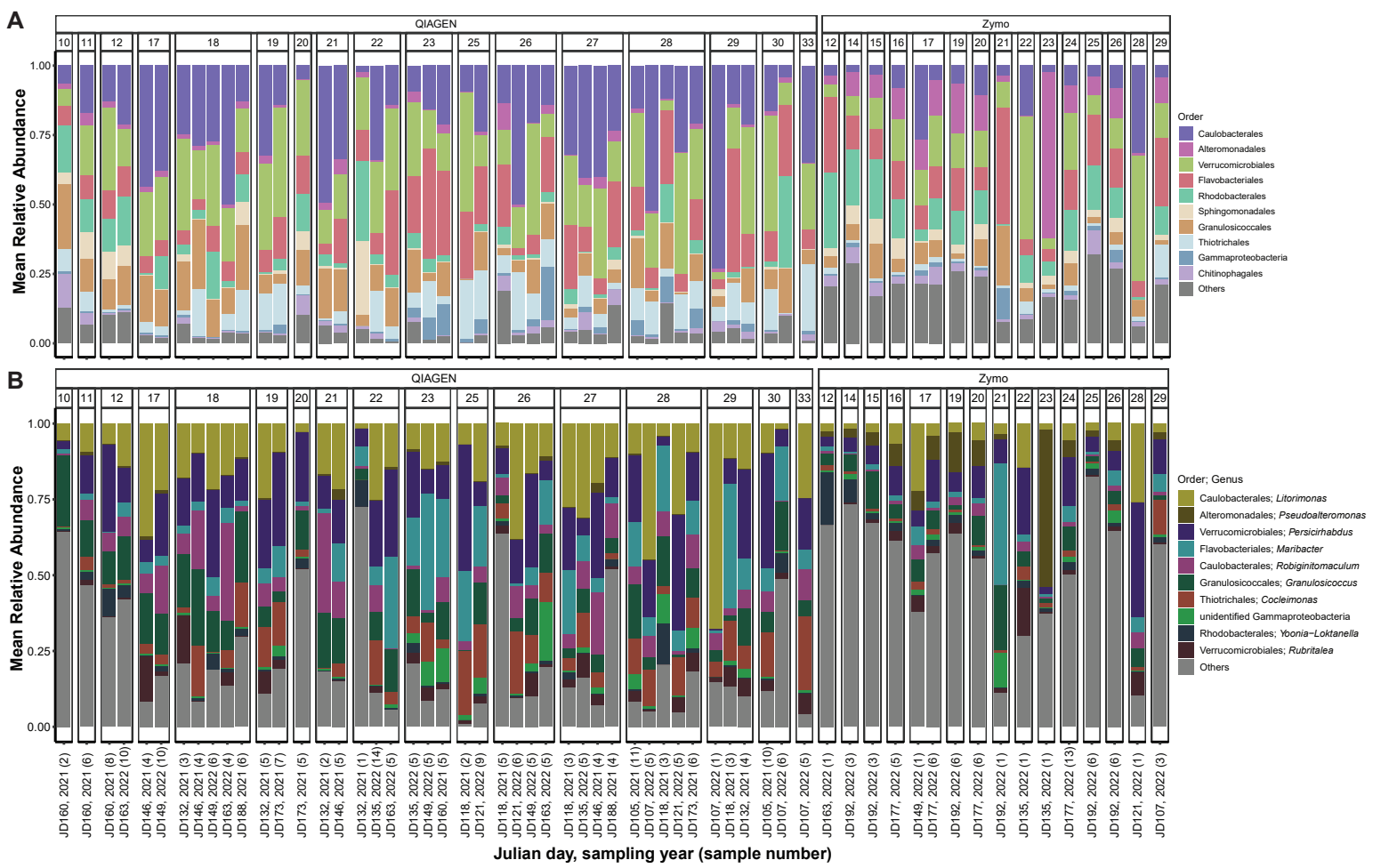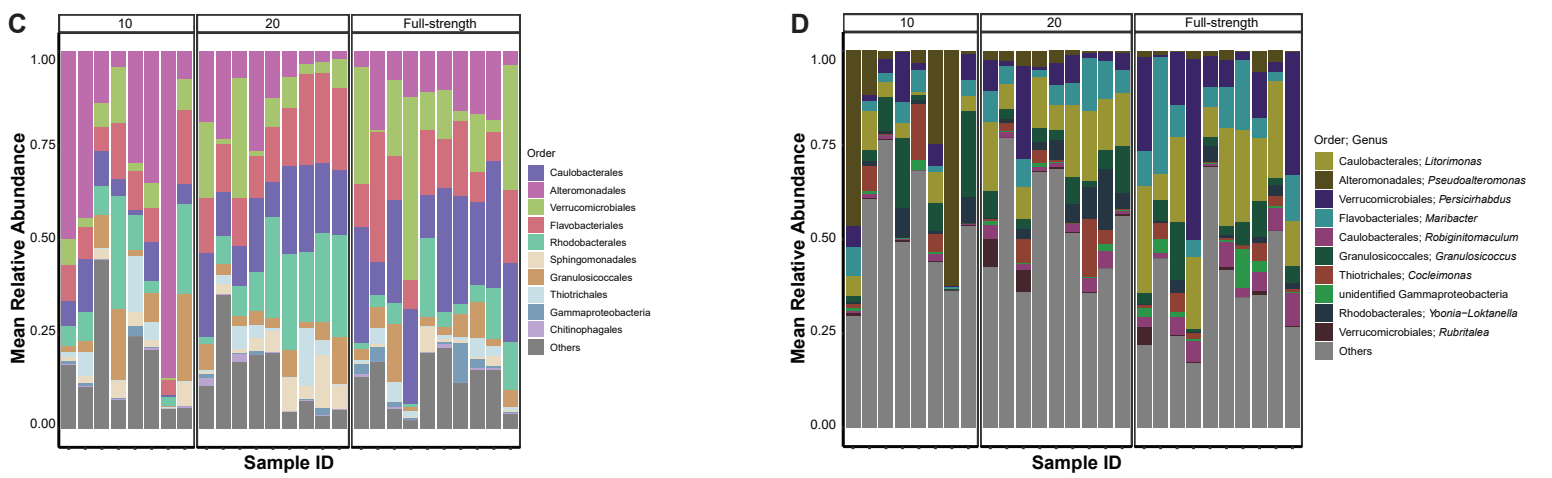

Supplement: Supplementary file 4 — Figure S4. The relative abundance of the 10 most abundant orders (A, C) or genera (B, D) in the field (A, B) and the relative abundance of these taxa in the lab samples (C, D). Field samples (A, B) are separated by extraction kit and salinity (facets) on the sampling day (x‐axis) and the per‐sample relative abundance is averaged across all samples from the same sampling day. Sample numbers are indicated in parentheses. All lab samples (C, D) are shown. Colous are consistent between the field and lab Day 4 plots. Tables include the output of models comparing the relative abundance of taxa in the field (Table S2) and lab Day 4 (Table S3). [file JPY-61-840-s010.pdf]
