# Supplementary material for: Abiotic stress destabilizes the bacterial community of sugar kelp, Saccharina latissima (Phaeophyceae)
Source: J Phycol. 2025 May 28;61(4):840–57. doi: 10.1111/jpy.70033 (PMC12351371; doi:10.1111/jpy.70033)
Supplement: Supplementary file 10 — Data S1. Supporting Information. [file JPY-61-840-s004.docx]

**Title**

Abiotic stress destabilizes the bacterial community of sugar kelp, *Saccharina latissima* (Phaeophyceae)^1^

**Author Information**

Siobhan Schenk^2^, [sschenk@mail.ubc.ca](mailto:sschenk@mail.ubc.ca), 604-827-1879

Biodiversity Research Centre, Department of Botany, University of British Columbia, 6270 University Blvd, Vancouver, British Columbia V6T 1Z4, Canada

Connor Glen Wardrop, [gwardrop@student.ubc.ca](mailto:gwardrop@student.ubc.ca)

Biodiversity Research Centre, Department of Botany, University of British Columbia, 6270 University Blvd, Vancouver, British Columbia V6T 1Z4, Canada

Laura Wegener Parfrey, [lwparfrey@botany.ubc.ca](mailto:lwparfrey@botany.ubc.ca)

Biodiversity Research Centre, Department of Botany and Zoology, University of British Columbia, 6270 University Blvd, Vancouver, British Columbia V6T 1Z4, Canada

Hakai Institute, PO Box 25039, Campbell River, British Columbia V9W 0B7, Canada

**Running Title**

Abiotic stress and kelp bacteria

# Abstract

As climate change progresses, the intensity and variability of freshwater outflow into the ocean are predicted to increase. The resulting increase in low-salinity events, paired with other abiotic stressors (including increasing temperatures), will be a source of stress for the kelp *Saccharina latissima* (*Saccharina* hereafter) and potentially *Saccharina*-associated bacteria. Bacteria influence host health and can facilitate or hinder host survival and acclimation to stressful abiotic conditions. Therefore, understanding how bacterial communities change under abiotic stress is critical to understand how abiotic stress will affect kelp physiology. We investigated the effect of abiotic stress on *Saccharina* and associated bacteria by surveying the bacterial community associated with *Saccharina* across naturally occurring salinity and temperature gradients, coupled with salinity manipulation experiments. Overall, *Saccharina* harbours a stable core bacterial community, which decreases in relative abundance under abiotic stress. In the field, both salinity and temperature shape the bacterial community, with temperature having higher explanatory power most of the time. In the lab, we confirm that the patterns observed in the field can be replicated by manipulating salinity alone. Decreased relative abundance of core bacteria and increased community dissimilarity in low-salinity in the lab, suggest that low-salinity alone can induce a stress response, detectable in the bacterial community of *Saccharina*.

**Key Index Words**

*Saccharina latissima,* Kelp*,* Bacteria, Core Microbiome, Salinity, Temperature, Abiotic Stress, Climate Change, Macroalgae

**Abbreviations**

UBC: University of British Columbia

IndVal: indicator species analysis

ENA: European Nucleotide Database

PERMANOVA: Permutational multivariate analysis of variance

AKP: Anna Karenina Principle

AIC: Akaike Information Criterion

BC: British Columbia

UK: United Kingdom

# Introduction

Kelp (*Laminariales*, brown algae) are marine foundation species that form dense underwater forests across the globe, providing food and habitat for other organisms (Steneck et al., 2002). Through ecosystem services and commercial harvest, kelp contribute an estimated $674 billion CAD per year to the global economy (Eger et al., 2023). However, kelp and the ecosystem services they provide are vulnerable to global change factors (Filbee-Dexter et al., 2019; Goldsmit et al., 2021) as are coastal organisms in general (Bindoff et al., 2019). Factors including increased coastal water turbidity (Filbee-Dexter et al., 2019), heatwaves (Smith et al., 2024), and lower ocean salinity (Davis et al., 2022; Filbee-Dexter et al., 2019; Andersen et al., 2011) have been associated with reductions in kelp recruitment, reproduction, and/or survival. As anthropogenic climate change progresses, higher temperatures are a global threat to kelp. Lower ocean salinity has received less attention but is predicted by modelling studies to be one of the most intense threats (Filbee-Dexter et al., 2019; Goldsmit et al., 2021), particularly in areas influenced by more variable and intense freshwater influx from glacial melt and variable snowpack melt.

There is a large body of evidence demonstrating that low salinity and high temperature are stressful to kelp. High temperatures and marine heat waves have led to loss and reduction of kelp populations in warmer areas, in some cases leading to long term extirpation (Starko, 2022; Smith 2024). Low salinity can have strong localized effects in melt and floodwater influenced areas. For example, field observations from Australia (Davis et al., 2022) and Norway (Andersen et al., 2011) found up to 100% mortality of kelp sporophytes after freshwater floods, followed by a re-growth of kelp in the following months. Lab studies also showed that low salinity is stressful for kelp across lifecycle stages. Research on the microscopic stages showed lower spore settlement and gametophyte germination rates (Lind & Konar, 2017) and lower production of sporophytes (Farrugia Drakard et al., 2025) in low salinity. Similarly, high temperatures negatively affect the microscopic stages across kelp species (Weigel et al., 2023; Becheler et al., 2022; Zhang et al., 2013). For the macroscopic sporophyte stage, lower photosynthetic efficiency (Bollen et al., 2016; Karsten et al., 2007), nitrogen uptake rates (Kumar et al., 2018), and growth rates (Mansilla et al., 2014) are reported in low-salinity treatments. High temperature also has negative effects on the macroscopic stage of multiple kelp species (Simonson et al., 2015).

The bacterial community of macroalgae can influence growth, morphology (Marshall et al., 2006; Provasoli & Pintner, 1980), and tolerance of abiotic conditions (Dittami et al., 2016). High temperatures have been shown to influence kelp-associated bacterial communities (Minich et al, 2018; Vadillo Gonzalez et al., 2024), but the influence of low salinity on the kelp-associated bacterial community is not fully understood. Studying how abiotic stressors influence the kelp-associated bacterial community, and whether these changes exacerbate or ameliorate kelp stress tolerance may provide additional insight into both the kelp response to stressors and the potential of microbial manipulation as a tool to promote resilience (Li et al., 2022).

Salinity is a strong determinant of bacterial community composition. In fact, a meta-analysis shows that host-associated bacterial community composition was primarily shaped by host association and salinity, even when other variables, including pH and temperature are taken into account (Lozupone & Knight, 2007). Field studies (van der Loos et al., 2023) and manipulative lab experiments (Saha et al., 2020; Stratil et al., 2014) showed that salinity is an important factor in shaping the bacterial communities on non-kelp algal hosts. Studies of kelp (Lemay et al., 2018; Weigel & Pfister, 2019; Davis et al., 2022) and the brown algae *Fucus distichus* (Davis, 2022) have shown that seasonal changes and site differences are also important factors shaping the bacterial community of brown algae. Perspective essays have highlighted the need for 1) time series in the field across multiple sites to disentangle the effects of abiotic factors, and 2) lab experiments that complement field studies to isolate the influence of particular abiotic factors on the bacterial community (Trevathan-Tackett et al., 2019) and on host condition. The need for paired lab and field studies is apparent in the kelp literature, as different studies have found strong influences of temperature or salinity. Field studies across sites at one time-point show that salinity, but not temperature, significantly alter the bacterial community associated with the kelp *Nereocystis* *luetkeana* (Weigel & Pfister, 2019). However, in the lab, high temperatures alter the bacterial community of the kelp *Ecklonia radiata* (Vadillo Gonzalez et al., 2024). We are not aware of lab-based studies that have examined how salinity alters the bacterial community of kelp.

Bacterial community composition typically changes in response to change in abiotic conditions across host-microbe systems, though the nature of these changes and whether they are associated with positive, negative, or neutral host outcomes varies across systems. In studies examining the bacterial community of hosts under non-stressful salinity gradients, including in green algae (*Ulva* sp.) from localities with different salinity (van der Loos et al., 2023) and transplanted seagrasses (Adamczyk et al., 2022), changes in the bacterial community composition were not associated with changes in host condition. For a freshwater strain of the brown alga *Ectocarpus sp.,* the bacterial community associated with the strain was essential to the alga’s ability to grow in fresh water (Dittami et al., 2016), showing that the bacterial community can improve the ability of brown algae to tolerate low salinity.

An important step in assessing the relationship between abiotic conditions, the bacterial community, and host condition is establishing whether the bacterial community is generally stable under the normal range of conditions and only exhibits major changes under stressful conditions. A stable host-associated bacterial community is a requirement for the Anna Karenina Principle (AKP) to potentially apply. AKP predicts that in stressful conditions beta diversity—the variation in community composition across samples—will increase (Zaneveld et al., 2017). Increased beta diversity indicates a less stable, less consistent bacterial community, which we refer to as destabilization herein. This destabilization may be a result of the disruption of the host filtering mechanisms that typically maintain a stable, low-diversity bacterial community. Host-filtering is a concept inspired by the environmental filtering metaphor that describes the environment as a selective filter that restricts the organisms that can establish and persist (Kraft et al., 2015). Host-filtering refers to the selective processes mediated by host characteristics that attract or repel microbial taxa and thereby shape the microbial communities associated with the host. For example, the host kelp produces polysaccharides that attract bacteria as a food source (Bengtsson et al., 2011; Sandbakken et al., 2018; Weigel et al., 2022) and produces chemical defences that deter bacterial colonizers, including reactive oxygen species (Egan et al., 2013; Saha & Weinberger, 2019) and halogens (Lavecchia et al., 2024; Tymon et al., 2017). This filtering is in line with the chemical “gardening” concept presented elsewhere (Saha & Weinberger, 2019). We predict that destabilization will be accompanied by an increase in alpha diversity and a decrease in the relative abundance of core taxa. These patterns have been observed repeatedly in marine systems on corals (McDevitt-Irwin et al., 2017) and occasionally on sponges (Pita et al., 2018) in response to abiotic stress.

An alternative pattern that is commonly observed is a decline in alpha diversity and a directional change in the bacterial community (turnover) in stressful conditions. A meta-analysis examining how temperature affects the bacterial community across a very wide range of taxa (aquatic and terrestrial) reveals consistent changes in community composition and that alpha diversity is more likely to decrease, while a general increase in beta-diversity was not observed (Li et al., 2022). Overall, evidence to date suggests that the bacterial community responses to abiotic stress vary across host organisms and that AKP patterns and destabilization may be characteristic of only a few host-microbe systems. More broadly, establishing causality in the link between host condition and change in host-associated bacterial communities in response to stress is an open challenge that requires experimental manipulation (Egan et al., 2013; McDevitt-Irwin et al., 2017; Pita et al., 2018). Field reciprocal transplant studies of corals across reef pools with different thermal profiles and a paired lab experiment (Ziegler et al., 2017) find long-term directional changes in the bacterial community (field results) that are better adapted to the temperature profiles of the environment experienced by the host and its bacteria (lab results). Another, lab-based study on anemone bacterial communities (Baldassarre et al., 2022) finds a similar pattern, where warm-adapted anemones are more resistant to heat stress than non-warm-adapted anemones and heat stress resistance is at least partially mediated by the bacterial community. Both studies controlled for the effect of host genetics and showed that host-associated bacterial communities changed directionally.

We conducted a two-year field survey starting in April (in 2021 and 2022) paired with a concurrent lab study in 2022 to isolate the effect of salinity on the bacterial community of the kelp *Saccharina latissima* ((L.) C.E. Lane, C. Mayes, L. D. Druehl, and G. W. Saunders) hereafter referred to as *Saccharina*. In our field study, we also incorporated local temperature in our analyses as a way to contextualize the importance of salinity compared to other abiotic parameters. Specifically, we tested if observed shifts in the bacterial community were consistent with 1) a shift to a distinct low salinity community (turnover) or 2) destabilization of a normally stable community, consistent with loss of host filtering. Evidence for destabilization requires first establishing community stability in non-stressful conditions. Existing data from European populations suggest that *Saccharina* has a relatively stable core bacterial community (King et al., 2023), and we tested for stability and the presence of a core community in non-stressful conditions here. We then tested for three patterns associated with for destabilization: increased community dissimilarity (beta diversity) in stressful abiotic conditions consistent with AKP, increased alpha diversity, and decreased relative abundance of the core community.

Our extensive dataset showed that the *Saccharina* bacterial community was largely stable with a consistent suite of core bacteria that are maintained across time, space, and salinity gradients. Layered on this broad pattern of stability, we found a small but statistically significant effect of salinity on the overall bacterial community composition in the lab and field. In the field, temperature generally had a stronger influence on the bacterial community than salinity. Together, these results suggest that high temperature and low salinity likely function as additive stressors for *Saccharina*.

# **Methods**

## Field site description

Five field sites near Vancouver, Canada were visited across two successive years (Figure 1A) during the time of the annual freshwater influx caused by snow melt in the Canadian Rockies in late spring, hereafter called the freshet. Both years, sites were sampled every two weeks (Figure 1B) at low tide, from April (April 15^th^ in 2021 and April 17^th^ in 2022) until July (July 7^th^ in 2021 and July 25^th^ in 2022); peak freshwater input and minimum salinity typically occur in June (Ryan et al., 2019; Rapaport, 2024). We selected sites based on the presence of *Saccharina* and their typical salinity profiles through the freshet (Ryan et al., 2019), aiming for two sites that maintained relatively high salinity (above 20 psu) and two that dropped to salinities stressful for kelp (10 psu—15 psu); sites are numbered 1–5 from lowest to highest salinity. The dominant freshwater source in our study is the Fraser River, with a smaller, much less significant source of freshwater from Indian Arm (Figure 1A). The dynamics of the Fraser River interacting with the Salish Sea are well-documented elsewhere (Ryan et al., 2019; Rapaport, 2024).

In 2021, we collected data at Site 1 (Lighthouse Park; 49.329°,-123.264°), Site 2 (Third Beach, Stanley Park; 49.302°,-123.158°); Site 3 (Sandy Cove Park; 49.333°,-123.222°), and Site 5 (Girl in a Wetsuit, Stanley Park; 49.304°,-123.126°; Figure 1A). Salinity dropped below 15 psu at three of the four sites (Site 1, Site 2, and Site 3; Figure 1C), so in 2022, we replaced Site 1 with Site 4 (Cates Park; 49.300°,-122.958°; Figure 1A), which maintained higher salinity through the freshet and led to a more balanced sampling design. Within a sampling event, North/West Vancouver sites (Site 1 in 2021 or Site 4 in 2022 and Site 3 for both years) were always sampled on the same day and the Vancouver sites (Site 2 and Site 5) were sampled the subsequent day due to the tide height required and travel time.

## Site conditions

In 2021 and 2022, we recorded water salinity (Figure 1C) and water temperature (Figure 1D) at the start of each site visit. In 2021, all measurements were conducted with an YSI ProQuatro Multiparameter Metre. On two sequential samplings in 2022, salinity only measurements were taken with a refractometer because the YSI was not available. In all cases, the measurement instrument was calibrated as per manufacturer’s instructions prior to each sampling and abiotic measurements were repeated three times per site visit (means used for data analysis).

## Field bacterial data collection

In both years, two water samples, two rock swabs, and at least six *Saccharina* swabs were collected during each site visit to capture changes in the bacterial community throughout the freshet.

We swabbed the bottom 10 cm of the *Saccharina* thallus (the meristem region) because it is the newest and most selective tissue (Lemay et al., 2021), presumably hosting the bacterial community most indicative of *Saccharina.* Samples were taken wearing gloves sprayed with 70% ethanol between samples. Rocks were sampled to capture the background biofilm communities, for which we selected regions roughly 5 x 5 cm that were free of visible organisms. We gently rinsed the *Saccharina* and rocks with 0.22-μm filter-sterilized seawater before swabbing the surface for 10 s with a cotton-tipped swab (VWR, CA10805-154). The swab was then broken off into a cryovial (VWR, CA66021-993). After taking bacterial samples, we recorded the presence of any blisters on the kelp thallus. Blisters were observed only once on June 10^th^, 2021 (Julian day 161), at Site 2 on three *Saccharina* individuals. Blistered tissue was not sampled.

Water column bacterial samples were collected by pre-filtering seawater through a
150-μm mesh before filtering the water through a 0.22-μm membrane (MilliporeSigma, Sterivex™ Filter Unit) until the filter clogged or 500 mL of water had been sampled. The Sterivex^TM^ was stored in a Whirl-Pak (VWR, 13500-390).

All samples were stored in a cooler with ice packs at -20°C until they could be brought to the lab (within three hours of sample collection), where they were then stored at -70°C until extraction.

## Lab experiment protocol

In 2022, we performed a manipulative lab experiment to isolate the effect of low-salinity stress on *Saccharina* (Figure 1E). We define stress as a condition that adversely affects growth via damage and/or resource allocation associated with damage prevention and cellular repair (Davison & Pearson, 1996; Harley et al., 2012). We performed eight experimental trials through the 2022 field season, corresponding to each field-sampling event (Figure 1B), to assess the influence of salinity over time. All *Saccharina* used in the experiments were collected from Site 5 because Site 5 has, by far, the largest population of *Saccharina* and salinity stays relatively high. This enabled us to test whether the community shifted in response to low salinity exposure. After collecting the bacterial samples in the field at Site 5 as described above, 18 *Saccharina* individuals (six of which were swabbed and are the 2022 field *Saccharina* samples from Site 5) were collected (the holdfast, stipe, and bottom 15 cm of the blade), numbered with flagging tape wrapped around the stipe, and transported in a cooler filled with seawater from Site 5 (approximately 1 h transit). We selected to place the *Saccharina* directly in the different salinity treatments rather than acclimating or ramping the salinity stress, as a meta-analysis of temperature manipulation studies found that acclimating or ramping attenuates the stress response (Li et al., 2022) and the goal of the lab study was to maximize stress.

*Saccharina* were incubated in seawater at 10°C on a 12 h light: 12 h dark photoperiod with bubblers to induce water motion. Each experimental trial included six 8 L aquaria (two per treatment), with three *Saccharina* meristems per aquaria (Figure 1E), in one of three salinity treatments: 10 psu, 20 psu, and unaltered sea water (full-strength hereafter). The low-salinity treatment (10 psu) represents the lowest salinity observed at our sites (Figure 1C) and is reported to be stressful as measured by significantly lower effective quantum yield in sporophytes (Karsten, 2007). Significant increased death and damage were observed between 6 psu and 11 psu for *Saccharina* germlings (Peteiro & Sánchez, 2012). A salinity of 20 psu is commonly experienced at our sites (Figure 1C) and was previously associated with differential gene expression compared to higher salinity (Monteiro et al., 2019). The full-strength seawater is pumped from 30 m depth in Burrard Inlet and brought to UBC by truck. The salinity fluctuated between 31 psu and 32 psu depending on the experimental trial. Salinity was lowered by adding deionized water to the full-strength seawater as described previously (Gerard et al., 1987; Peteiro & Sánchez, 2012) and salinity was checked with a refractometer.

Most experimental trials lasted four days with samples taken on Day 0 (in the field), Day 1 (24 h after collection), and Day 4. Our first experimental trial lasted six days (Figure 2A). On Day 6, the *Saccharina* in 10 psu salinity died (turned green and fell apart to the touch; Figure 2A), so we shortened the incubation time to three days for experimental trial two. We did not observe significant *Saccharina* damage on Day 3, so we extended the experimental duration to four days for the remaining experimental trials (Figure 2A). For experimental trial five, there are no dry weight measurements on Day 4 because we extended the experimental time to fourteen days after collecting all other Day 4 samples, to observe whether the kelp thallus sections in the 10 psu salinity would become brittle to the touch as occurred in trial one. They did not.

## Lab bacterial collection protocol

We collected one sample of the *Saccharina* microbial community, one of aquaria water, and one of aquaria substrate per aquaria for two replicate aquaria for each salinity treatment (6 total aquaria) on Day 1 and Day 4 for each experimental round, and we repeated the experiment eight times throughout the freshet (Figure 1E). All lab microbial sampling followed the same protocol in the field with the following modifications: 1) substrate samples were taken by swabbing the airline tubing of the bubbler rather than rocks, 2) aquaria water was not pre-filtered with a sieve before taking bacterial samples, and 3) lab *Saccharina* samples (Day 1 and after) were swabs of all three kelps in the same aquaria to avoid pseudo-replication; we assumed the bacterial community was homogenized within the same aquarium because microbial changes in kelp incubated with other macroalgae have been previously observed (Chen & Parfrey, 2018). *Saccharina* individuals used in the experiment were all collected from Site 5. Six of the 18 individuals in the lab experiments were the individuals swabbed as part of the Site 5 field data (Day 0). These six individuals were evenly distributed across treatments such that each aquaria contained one *Saccharina* individual swabbed on Day 0 and two individuals that had not been swabbed. We note that prior swabbing may have altered the *Saccharina* microbial community, though would not be expected to differ by salinity treatment because all treatments were handled equivalently. We only present results of Day 4 samples for simplicity.

## Lab *Saccharina* condition

After taking bacterial samples, the condition of each *Saccharina* was assessed on an individual basis (n=6 per salinity treatment/time point/experimental trial) by quantifying blistering occurrence, wet weight, estimated quantum yield, and ratio of dry weight to wet weight on the last day of each experimental trial (Figure 1E). These measurements served as a phenotypic read-out to test whether the biologically relevant salinity treatments used here induced stress, according to the definition presented above (Davison & Pearson, 1996; Harley et al., 2012).

Blistering occurrence was recorded as in other kelp studies (Davis et al., 2022; Qiu et al., 2019). Estimated quantum yield measurements were taken with a Junior-PAM Chlorophyll Fluorometer as described in the user manual. Briefly, we calibrated the PAM and dark acclimated the *Saccharina* for five minutes on a damp cloth. Then, we placed the optical fibre over the thallus base (just above the stipe) and took the measurement. Wet weight measurements (holdfast, stipe, blade section) were taken after blotting dry the *Saccharina,* and the kelp were promptly returned to aquaria after weighing.

Dry weights were obtained as follows. After taking wet weight measurements on the last experimental day, stipe and holdfast were cut off from the blade section (Bollen et al., 2016). We weighed the blade sections before drying them in at 60°C for at least 24 h, and reweighed them (Bollen et al., 2016; van Ginneken, 2018).

## DNA extraction and sequencing

In 2021, all DNA was extracted with QIAGEN DNeasy PowerSoil Pro Kit 96-well plate; 47017 or single tube; 47014 following the manufacturer’s protocol. In 2022, 71% of field swabs (*Saccharina*, water, and rock) samples were extracted with QIAGEN 96-well kits. The remaining 29% of field samples were extracted with ZymoBIOMICS 96 MagBead DNA/RNA Kit (Zymo, R2136). All lab samples (experiments conducted in 2022) were extracted with the Zymo kit. QIAGEN PowerSoil Max beads (QIAGEN, 12988-10) were used for the initial bead-beating step, and then the Zymo kit was used following the manufacturer’s protocol. The extraction method for each sample is indicated in the microbial metadata. The change was due to global supply chain issues. Each extraction plate included a blank swab or Sterivex^TM^ membrane (extraction blank, n=12).

Primers for PCR were 515F (5’-GTGYCAGCMGCCGCGGTAA-3’) and 806R (5’-GGACTACNVGGGTWTCTAAT-3’), which target the V4 region of the 16S rRNA gene. Both primers have an Illumina adaptor and golay barcode on the 5’ (Parada et al., 2016; Quince et al., 2011). PCR reactions were as follows: 1 μL extracted DNA (for extracted samples) or water (for PCR blanks, 1 per PCR plate, n=12), 15 μL Phusion™ High-Fidelity DNA Polymerase (Thermo Fisher, F530S), 2.4 μL of forward primer, 2.4 μL reverse primer (primers are from a 2.5 μM working stock solution), to a final reaction volume of 30 μL with molecular water (Thermo Fisher, SH30538FS). PCR reactions consisted of 30 s initial denaturation at 98°C, 25 cycles of amplification (30 s at 98°C, 30 s at 55°C, and 20 s at 72°C), and a final elongation step at 72°C for 10 min, which are the conditions recommended for the Phusion^TM^ polymerase. Samples without a visible band on a DNA gel after two attempts with 25 cycles were amplified with 35 cycles (indicated in the microbial metadata).

PCR reactions were cleaned with the QIAGEN QIAquick PCR Purification Kit (28104 or 28181), and quantification was performed with the Quan-IT PicoGreen assay kit (Thermo Fisher, P7589) following the manufacturer’s protocol. Samples were pooled to equal concentrations and sent for Bioanalyzer at the UBC Sequencing Facility, Vancouver, British Columbia. In 2021, the library was sent for Illumina MiSeq at the Hakai Institute, Campbell River. In 2022, both libraries were sent for illumina MiSeq at the UBC Sequencing Facility. In all cases, libraries were constructed with the Illumina, MS-1023003 kit (MiSeq v3, 2x300).

## Sequence data processing

Raw, demultiplexed reads were downloaded from the Illumina hub and imported into RStudio (R version 4.2.2, RStudio v2022.10.31; (Posit team, 2022; R Core Team, 2022)). Reads were processed following the dada2 pipeline (v1.24.0; Callahan et al., 2016). Primers were removed; reads were truncated to maintain high quality for downstream analysis with the filterAndTrim function (275 bp for forward and 200 bp for reverse in 2021 and 230 bp for forward and 175 bp for reverse in 2022); error rates were calculated; paired forward and reverse reads were merged; a sequence table was constructed; and chimeric reads were removed. The 2021 and 2022 sequence tables were merged (mergeSequenceTables) and amplicon sequence variants (ASVs) that differed by only the end base pair were collapsed (collapseNoMismatch), prior to assigning taxonomy with SILVA v138 (McLaren, 2020) formatted for the dada2 pipeline (McLaren & Callahan, 2021).

The merged sequence table, the taxonomy table, and metadata were grouped into a phyloseq object for filtering (v1.40.0; McMurdie & Holmes, 2013). Unassigned taxa and taxa assigned to chloroplasts, mitochondria, or eukaryotes were removed in addition to sequences assigned as *Pseudomonas*. *Pseudomonas* were present at high relative abundance in multiple extraction blanks and was abundant in nearly all samples extracted with the Zymo kit, but present in only 1 sample extracted with the QIAGEN kits across both years; *Pseudomonas* is very likely a Zymo kit contaminant. Next, samples with fewer than 1000 reads were removed; then, ASVs representing less than 0.001% of total reads in the dataset were removed; counts in the ASV table that were five or less were converted to zero (per sample filtering, to minimize the effect of barcode switching); and ASVs found in less than two samples were removed. Finally, all samples from one 10 psu salinity aquarium in experimental trial five were removed (three Day 4 samples total) because we noticed that there was *Desmerestia viridis* lodged in the holdfast. *Desmerestia sp.* produce sulfuric acid (Eppley & Bovell, 1958) and pH alters the bacterial community of kelp (Qiu et al., 2019). We also removed the last field sampling event in 2022 (July 26^th^ and 27^th^; Julian day 206 and 207) to make the time covered in 2021 and 2022 consistent as our sampling ended earlier in 2021. We retained the data from the lab experiment started on July 27^th^, 2022 (Julian day 207). In total, filtering retained 17,438,672 of 24,862,382 total paired reads, 3,040 of an initial 29,105 ASVs, and 713 of the initial 1,038 samples, with a mean of 21,432 reads per sample. See Table S1 for final sample number by year and site (field) or sample type (lab). We converted the data to an iNEXT (v3.0.0; Hsieh et al., 2022) compatible format with the metagMisc package (v0.0.4; Mikryukov, 2022) to perform coverage-based rarefaction. The sample coverage was set to 0.8 and iterated 1000 times.

We tested the influence of DNA extraction kit on diversity. By Kruskal-Wallis test comparing field *Saccharina*, rock, and water samples followed by a Benjamini-Hochberg correction for multiple comparisons, *Saccharina* (χ_1_^2^ = 120.07, p<0.001) and rock samples (χ_1_^2^ = 25.465, p<0.001) extracted with the Zymo kit had higher richness than those extracted with the QIAGEN kit, but not water samples (χ_1_^2^ = 2.64, p=0.10). We tested for differences in beta diversity using PERMANOVA comparing samples extracted with Zymo versus QIAGEN for the same sample type and at a similar time of year and we found significant differences between extraction kits for *Saccharina*, rock, and water samples, indicating that the different extraction kits capture different bacterial communities. Therefore, we accounted for extraction kit in all analyses that include samples extracted with different kits (2022 field samples).

## Statistical analysis

To compare blistering incidence between salinity treatments, we used a Fisher’s exact test with a Benjamini-Hochberg correction with the package rstatix (v0.7.1; Kassambara, 2022b).

For all field bacterial data analyses, we included comparable dates: Julian day 105 to 189 in 2021 and Julian day 107 to 193 in 2022. In cases where we include temperature in our analysis, the days with no water temperature (Julian day 143-164 in 2022) are excluded.

We ran permutational analyses of variance (PERMANOVA) on rarefied data with the adonis2 function in the package vegan (v2.6-4; Oksanen et al., 2022) with the distance metric set to Bray-Curtis. Marginal PERMANOVAs were run the same way, adding the by=“margin” argument. In all cases, we tested for equal dispersion with the betadisper function (equal unless stated otherwise) and ran post-hoc Pairwise Adonis tests in the pairwise.adonis package (v0.4.1; Arbizu, 2017).

We calculated the Shannon-Wiener diversity index with the diversity function in package vegan (Oksanen et al., 2022). The Bray-Curtis dissimilarity index between samples was calculated with the divergence function in the package microbiome (v1.22.0; Lahti & Shetty, 2012) as in Lesser et al. (2016).

To compare means between groups, we ran an analysis of variance (ANOVA) followed by a Tukey post-hoc test. In all cases, we tested the assumption of equal variance between groups with Levene’s test in the package car (v3.1-1; Fox & Weisberg, 2019) and validated the assumption of normality with QQ plots. When the assumption of normality was violated, we ran a Kruskal-Wallis test followed by a post-hoc Wilcoxon test (R Core Team, 2022).

To identify the core *Saccharina* ASVs, we selected all field samples collected at salinity 20 psu or greater and ran an indicator species analysis (IndVal) on non-rarefied data with the function multipatt from the package indicspecies (v1.7.12; Caceres & Legendre, 2009) with 999 permutations, comparing substrate types (*Saccharina*, water, and rock). We used a threshold of 0.7 IndVal statistic, which required ASVs to be enriched and highly prevalent in *Saccharina* samples compared to other sample types (water and substrate samples). To calculate the relative abundance of core ASVs across temperature and salinity gradients, we ran linear regression models. We selected the best model by backwards Akaike Information Criterion (AIC) and tested the linear regression assumptions with plots (QQ and residuals vs fitted).

Taxa summary plots were generated by calculating the 10 taxa with the greatest relative abundance across all *Saccharina* samples in the field at the order and genus level. We ran linear regression models for each taxa identified as part of the top 10 most abundant taxa in the taxa plot to check for trends in relative abundance along the temperature and/or salinity gradients. We corrected for multiple comparisons with a Benjamini-Hochberg correction (R Core Team, 2022).

All plots were made with the ggplot2 (Wickham, 2016) and ggh4x (v0.2.3; Brand, 2022) packages and were saved as pdf files with ggpubr (v0.5.0; Kassambara, 2022a). Text modifications for plot labels were made with the package stringi (v1.7.12; Gagolewski, 2022).

## Reanalysis of published data

Raw data from King et al. (2023) were downloaded from ENA (PRJEB50679) and <https://doi.org/10.6084/m9.fgshare.19453889.v1>, processed with the dada2 pipeline, and filtered as described above and in their original publication. Core *Saccharina* ASVs were identified as those with a prevalence ≥ 0.8, replicating methods in King et al. (2023), as there are no comparison environmental samples available.

# Results

## Factors shaping the bacterial community of *Saccharina* in the field.

We examined how salinity shapes bacterial community composition on *Saccharina* and in the surrounding environment in the field in comparison to temperature. The bacterial community on *Saccharina*, rocks, and water differed significantly (PERMANOVA; Figure S1A). Both salinity and temperature varied over time and across sites (Figure 1C,D), and salinity was negatively correlated with temperature, and both salinity and temperature were correlated with seasonality, as measured by Julian day (Figure S2). Thus, we ran a marginal PERMANOVAs nesting by extraction kit to assess the unique explanatory power of salinity and temperature on *Saccharina*, water, and rock separately (Table 1), as well as NMDS plots to visualize the patterns (Figure S3A-F). We did not include Julian day as a variable in the model to avoid overfitting. Salinity and temperature were both significant and explained 1.3% and 1.4% respectively of variation in the bacterial community of *Saccharina* for QIAGEN-extracted samples (Table 1; Figure S3A,D). Temperature, but not salinity, is a significant explanatory factor for Zymo-extracted samples (Table 1; Figure S3A,D). For water samples, temperature and salinity showed the same patterns as for *Saccharina* but explained more unique variation in community composition (Table 1; Figure S3C,F). The bacterial community composition on rocks was not significantly influenced by temperature or salinity (Table 1; Figure S3B,E).

## Testing the influence of low salinity on *Saccharina*

We tested the influence of salinity on *Saccharina* and associated bacteria using replicated experiments throughout the freshet by incubating field-collected *Saccharina* in one of three salinity treatments: 10 psu, 20 psu, and full-strength (31–32 psu; Figure 1E). These are biologically relevant salinity levels for *Saccharina* that are within the range of normal variation experienced at our sites (Figure 1C) and were expected to differentially induce stress. We measured four stress-associated kelp phenotypes to verify that these salinity treatments did indeed differentially induce stress in *Saccharina*. We compared blistering incidence on Day 4 by Fisher’s Exact Test followed by a pairwise Fisher post-hoc test, which showed that our lowest salinity treatment had significantly greater blistering incidence than the other salinity treatments (Figure 2A). Experimental trial one lasted six days and experimental trial two lasted three days. We did not observe any blistering on the last day of these trials. However, on Day 6 of trial one, all *Saccharina* in the 10 psu salinity were green and disintegrated when touched, indicating death (Figure 2A). This was not observed again throughout the experiment, even when we extended experimental trial five to fourteen days, highlighting the importance of adequate biological replication.

We used ANOVAs followed by Tukey Post-Hoc tests for the other comparisons. The 10-psu salinity treatment had a significantly lower dry weight to wet weight ratio compared to the other salinity treatments (Figure 2B). The ratio of wet weight on Day 4 compared to Day 0 did not differ by salinity treatment (Figure 2C). Estimated effective quantum yield on Day 4 trended lower in the low salinity treatment but the differences between treatments were not significant (Figure 2D). These results suggest that *Saccharina* accumulated moisture but not additional biomass in low salinity and remained alive for the duration of the experiment.

## The effect of salinity on bacterial communities in the lab.

The bacterial communities on *Saccharina*, water, and substrate differed significantly from each other on Day 4 (PERMANOVA; Figure S1B). After establishing that our biologically relevant salinity treatments were stressful for *Saccharina* (Figure 2), we assessed the total influence of salinity on the bacterial community composition in the lab experiment using a PERMANOVA (Table 2) and visualized the differences by salinity treatment with NMDS plots for each sample type (Figure S3 G-I). Bacterial community composition on *Saccharina* and in the aquarium water differed significantly by salinity treatment, while there was no difference by treatment for substrate (Table 2; Figure S3G-I). Salinity treatment explained a larger amount of variation in water samples compared to *Saccharina* (Table 2; Figure S3G,I), following the same trend in explanatory power observed in the field (Table 1). We also saw a strong effect of experimental trial for *Saccharina* and water, indicating that the overall effect of salinity in the lab was robust to different starting bacterial communities (Table 2).

## Assessing the stability of dominant taxa.

We assessed the stability of the dominant taxa (order and genus) within *Saccharina* bacterial community across the salinity gradient visually and quantitatively. Plotting the 10 most abundant taxa on *Saccharina* highlighted the stability of the *Saccharina* bacterial community over the salinity gradient, as much of the community was consistently present (Figure S4, Table S2, Table S3).

To assess whether these taxa significantly changed in the relative abundance over the salinity and temperature gradients in the field, we ran linear regression models followed by a Benjamini-Hochberg correction (Table S2). At the order level (Figure S4A; Table S2) there were no significant changes in relative abundance across the salinity or temperature gradient for either extraction kit. At the genus level (Figure S4B; Table S2), *Litorimonas* (Caulobacterales) decreased with higher temperatures in QIAGEN-extracted samples, whereas in the Zymo-extracted samples *Pseudoalteromonas* (Alteromonadales) increased relative abundance in lower salinity and higher temperature (Figure S4B; Table S2). These results showed general stability in the relative abundance of dominant orders and genera across temperature and salinity gradients on *Saccharina* in the field.

We tested for differences in relative abundance of dominant genera and orders across salinity treatments in the lab with ANOVAs. At the order level (Figure S4C; Table S3), Caulobacterales was significantly less relatively abundant in the 10 and 20 psu treatments compared to full-strength salinity. A similar pattern was observed at the genus level (Figure S4D; Table S3), where *Litorimonas* and *Robiginitomaculum* (both Caulobacterales) were significantly less relatively abundant in the lower salinity treatments. These findings, paired with the field data, suggest that some Caulobacterales decreased in relative abundance under stressful abiotic conditions.

## Identifying core ASVs associated with *Saccharina*.

We identified the core bacterial community on *Saccharina* in the field when the salinity was relatively high (20 psu or greater). We excluded lower salinity samples when defining the core because these conditions may be stressful to the *Saccharina* populations studied here based on the results of the lab experiments (Figure 2). We used indicator species analysis (IndVal) with a threshold of 0.7 IndVal statistic value to identify ASVs that were enriched on field *Saccharina* compared to water and rock and at high frequency on *Saccharina*. We identified seven core ASVs (*Persicirhabdus* sp. ASV1, *Litorimonas* sp. ASV3, *Maribacter* sp. ASV4, *Cocleimonas* sp. ASV5, *Granulosicoccus* sp. ASV6, *Robiginitomaculum* sp. ASV7, Gammaproteobacteria ASV15; Table S4).

Then, we investigated the distribution of core bacteria ASV on *Saccharina* across much larger spatial scales by comparing our data to a study performed in the United Kingdom (UK) from September and August 2015 (King et al., 2023). The UK study did not include environmental comparison samples, so core bacteria were defined solely based on a prevalence of 0.8 or higher; this resulted in 25 core ASVs (Table S4).

Comparing the core ASVs between the two datasets, five of the seven core ASVs detected in our BC high salinity population were also found in association with *Saccharina* in the UK (*Litorimonas* sp. ASV3, *Maribacter* sp. ASV4, *Granulosicoccus* sp. ASV6, *Robiginitomaculum* sp. ASV7, Gammaproteobacteria ASV15), with ASV7 being a core ASV in both datasets (Table S4). Thirteen of the twenty-five core ASVs in the UK dataset were present in our *Saccharina* samples, though all except for ASV7 were at low prevalence (Table S4). Overall, these comparisons showed differentiation in the core bacterial community ASVs associated with *Saccharina* between the UK and BC, with some overlap.

## Assessing bacterial community change - Turnover.

We asked whether changes in the bacterial community associated with *Saccharina* across salinity gradients were consistent with a shift to a distinct low-salinity community (turnover) or destabilization of the community. First, we tested for turnover in the bacterial community of *Saccharina* by asking if there was a distinct *Saccharina* bacterial core community in low-salinity conditions in the field or the low salinity lab treatment (Figure 3; Table S4). We used indicator species analysis with the 0.7 IndVal threshold on field *Saccharina* samples collected when salinity was below 20 to identify the low-salinity core community found in the field. We found that the low salinity core on *Saccharina* in the field samples included all seven ASVs that were core on high salinity field samples, plus six additional ASVs found at a prevalence of 50-75% in high salinity samples (Figure 3; Table S4). These results indicate that differences in community composition were due to changes in relative abundance, rather than a loss or gain of ASVs across the salinity gradient in the field. This pattern was inconsistent with turnover.

The persistence of the *Saccharina* core community across the salinity gradient was supported by lab data showing that all core ASVs were maintained in the lab in all salinity treatments (Figure 3; Table S4). The average prevalence of core ASVs (core in both high and low salinities in the field) was 94% across all lab salinity treatments, while the prevalence of low-salinity core ASVs averaged 77% across all lab treatments (Figure 3; Table S4). The lab experiment allowed us to verify that changes in salinity can drive the patterns observed in the field, since salinity was highly correlated with temperature in the field (Figure S2). Overall, these data paint a picture of a stable bacterial community on *Saccharina* characterized by distinct core bacteria that persisted across abiotic gradients, with no evidence of turnover to a distinct and consistent low-salinity community.

## Assessing bacterial community change - Destabilization.

Next, we tested our prediction that destabilization occurs under abiotic stress by testing for reduced abundance of the core bacterial community and increased dissimilarity and alpha diversity in stressful conditions. Destabilization requires a stable host-associated bacterial community under non-stressful conditions, which was the case with *Saccharina*.

### Relative abundance of core ASVs

We ran linear regression models to quantify the relationship between the total relative abundance of the seven high salinity *Saccharina* core ASVs in the field and salinity (Figure 4A) and temperature (Figure 4B). The best linear regression model (by AIC) included both salinity and temperature. However, when comparing the significance of each term, we found that salinity was significant for the QIAGEN-extracted samples, but not the Zymo-extracted samples. Temperature was not significant for either kit (Figure 4A,B).

In the lab, where we can isolate the effect of salinity, we find that the total relative abundance of the core is lower in the 10 and 20 psu treatments compared to the full-strength treatment (ANOVA; Figure 4C). This shows that the core remains even in stressful low salinity conditions, but comprised a lower proportion of the overall bacterial community abundance in both the field (QIAGEN only) and in the lab. The lower relative abundance of the core observed on *Saccharina* both in the lab and field (Figure 4) is consistent with our predictions for community destabilization in response to salinity stress.

### Alpha-diversity

We used linear regression models followed by AIC model selection to assess the relationship between salinity and temperature with alpha diversity (Figure 5). We compared the results from *Saccharina*-associated bacterial communities to community patterns in the surrounding environment to determine whether the trends observed are unique to *Saccharina* (live host) or common across all microbial communities. The best model for alpha diversity of the field *Saccharina* samples includes temperature but not salinity (Figure 5A, Table S5), indicating that temperature has a larger influence on alpha diversity than salinity in the field. For the Zymo kit only, there is a significant effect of temperature, however, this should be interpreted with caution as the Zymo-extracted samples include few time points (Figure 5A, Table S5). The best model for the rock samples includes only the extraction kit, showing no pattern in alpha diversity by salinity or temperature (Figure 5B, Table S5). The best model for water samples included salinity but not temperature, and the QIAGEN-extracted water samples showed increased diversity in lower salinity (Figure 5C, Table S5).

In the lab, there was no difference in alpha diversity across salinity treatments for any sample type, as assessed by ANOVA (Figure 5D,E,F, Table S5). Overall, there was no evidence of a significant relationship between salinity and the alpha diversity of the *Saccharina* bacterial community.

### Community dissimilarity

We repeated the same analyses to assess changes in dissimilarity. Dissimilarity was measured within the same experimental unit (single field site and sampling day or lab salinity treatment and trial day). Low dissimilarity means that variation between samples was low (stable community). Under destabilization, we predicted increasing dissimilarity with increasingly stressful conditions.

The best model for field *Saccharina* included temperature only, and for QIAGEN-extracted samples, higher temperatures were significantly associated with higher dissimilarity (Figure 5G, Table S5). The best model for the rock samples included the extraction kit only, again with no pattern by abiotic conditions (Figure 5H, Table S5). The best model for the water samples included temperature and salinity, and for QIAGEN-extracted samples higher temperatures were significantly associated with lower dissimilarity (Figure 5I, Table S5).

In the lab, the dissimilarity of the *Saccharina* bacterial communities in the 10 psu treatment was significantly higher compared to the 20 psu and full-strength salinities (Figure 5J, Table S5). There was no significant difference by salinity treatment in the water or substrate communities (Figure 5K,L, Table S5).

Overall, in the field data, temperature was better than salinity at explaining dissimilarity between samples. The direction of the trend differed between *Saccharina* (Figure 5A,D) and water (Figure 5C,F) samples, and only the results for *Saccharina* were consistent destabilization in stressful conditions. Similarly, dissimilarity in the lab was higher at low salinity (10 psu) for *Saccharina* only (Figure 5J), consistent with destabilization.

# Discussion

The predicted (Filbee-Dexter et al., 2019) and ongoing die back of kelp forests (Christie et al., 2019; Davis et al., 2022) due to climate change represents a global concern because of the environmental, economic, and cultural value that these foundation species provide (Bindoff et al., 2019; Eger et al., 2023). We find that salinity influences the bacterial community of *Saccharina,* in agreement with other macroalgal studies in the lab (*Agarophyton*; Saha et al. (2020) and *Fucus*; Stratil et al. (2014)) and in the field (*Ulva*; van der Loos et al. (2023) and *Nereocystis*; Weigel & Pfister (2019)).

A change in the bacterial community composition can influence host health in a variety of different ways. In some cases, a short-term change in the bacterial community allows the host to better tolerate stressful abiotic conditions (high temperatures, Baldassarre et al., 2022; Ziegler et al., 2017). Heat stress in the kelp *Ecklonia* results in bacterial community turnover, but no interaction between the bacterial community and the host stress response (Vadillo Gonzalez et al., 2024). In other cases, host-associated bacteria are variable across non-stressful conditions including localities (the seagrass *Zostera marina*, Adamczyk et al., 2022) and natural salinity gradients (*Ulva* sp*.*, van der Loos et al., 2022). In other cases, a destabilization of the bacterial community—characterized by increased beta diversity and declining abundance of characteristic taxa (core taxa)—is associated with host stress. A destabilized bacterial community associated with host stress (usually high temperature) has been observed in corals (McDevitt-Irwin et al., 2017) and on some sponges (Pita et al., 2018).

Our lab experiments at biologically relevant salinity levels (Figure 1) show that low salinity induced stress in *Saccharina* (Figure 2), which allowed us to investigate the bacterial community changes associated with stress, and to ask whether the community changes observed in low salinity were the results of turnover or destabilization. In the case of turnover (option 1), we expected to see a new, stable, bacterial community in low salinity. In the case of destabilization (option 2), we expected increased community dissimilarity (as predicted by AKP, Zaneveld et al., 2017) along with a reduction of core bacteria and increased alpha diversity. These patterns are consistent with decreased host filtering in stressful conditions.

Our data supports option 2: *Saccharina*-associated bacterial community was destabilized in low-salinity conditions. In both the lab and the field, the relative abundance of core ASVs was lower in lower salinity (Figure 4). These high-salinity core ASVs were not replaced by a distinct and consistent low-salinity community (Figure 3). At the genus and order level, we found that most taxa were present at similar relative abundances at all salinities, indicating a stable community with no clear evidence of turnover (Figure S4, Table S2, Table S3). Additionally, our lab data showed increased dissimilarity in low salinity (Figure 5), supporting the AKP prediction (Zaneveld et al., 2017). In the field, we also observed increased dissimilarity, but this is better explained by high temperature than low salinity. We found no association between alpha diversity and salinity (Figure 5), showing that increased dissimilarity was not explained by increased alpha diversity.

Our lab experiments used *Saccharina* from a relatively high salinity site and tested the influence of low salinity. Future studies shifting *Saccharina* from low salinity into high salinity conditions are needed to determine whether these changes are reversible and observed across populations. Furthermore, determining whether these changes in the bacterial community mitigate or exacerbate the effects of low salinity stress on kelp requires further experimental manipulation.

Our study highlights the importance of pairing field observations, which provide a more holistic view of the system, with lab experiments, which permit the manipulation of a single variable (salinity) without confounding effects of other covariates present in the field (as suggested by Trevathan-Tackett et al., 2020). Here, salinity was strongly correlated with water temperature (Figure S2) during the freshet in the Fraser River Estuary and overall, temperature appeared to have the strongest influence the bacterial community of *Saccharina* in the field compared to salinity. Temperature explained slightly more variation in bacterial community composition than salinity, though both were significant factors (Table 1; Figure S3). Temperature was a significant predictor of community dissimilarity in the multivariate model assessing the relationship between dissimilarity and abiotic stress (Figure 5), but salinity was not. The lab experiment conducted at local biologically relevant salinity levels (Figure 1) allowed us to conclude that salinity can alter the *Saccharina* bacterial community (Table 2, Figure S3) and that low salinity can increase community dissimilarity (Figure 5). Together, these results suggest a common pattern of destabilization in the bacterial community associated with *Saccharina* in response to abiotic stressors. These findings provide a foundation for manipulative studies that are necessary to determine whether stress-induced destabilization of the kelp microbiome mitigates or exacerbates the effects of stress on the kelp host.

# Data Availability

Raw reads for this study are available on the European Nucleotide Database (ENA) under project PRJEB60884. All code and metadata are available on Borealis <https://borealisdata.ca/dataset.xhtml?persistentId=doi:10.5683/SP3/ILQ9UJ>.

# Contributions

Siobhan Schenk: conceptualization (equal), data collection (equal), writing (equal), editing (equal), data analysis (equal).

Connor G. Wardrop: data collection (equal), editing (equal).

Laura W. Parfrey: conceptualization (equal), data collection (equal), writing (equal), editing (equal), data analysis (equal).

# Acknowledgements

We thank E. Adamczyk, G. Ainsworth-Cruickshank, V. Billy, O. Moss, and V. Supratya for their help with fieldwork, B. Segovia and G. Lajoie for their help with data analysis, R. d’Entremont for editing the latest version of the manuscript, the UBC staff who helped us with the lab experiment, in particular J. Trat and J. Ng, and Hakai for their expertise with Illumina Next Generation Sequencing.

# Funding

Siobhan Schenk: Ocean Leaders Fellowship, British Columbia Graduate Fellowship. Siobhan Schenk and Connor G Wardrop: University of British Columbia Funding. Laura W Parfrey: NSERC and Tula Foundation.

# Conflict of Interest

The named authors have no conflict of interest, financial or otherwise to report.

# ORDCID

Siobhan Schenk 0000-0003-0572-9303

Laura W Parfrey 0000-0001-6959-7616

# References

Adamczyk, E. M., O’Connor, M. I., & Parfrey, L. W. (2022). Seagrass (*Zostera marina*) transplant experiment reveals core microbiota and resistance to environmental change. *Molecular Ecology*, *31*(19), 5107–5123. https://doi.org/10.1111/mec.16641

Arbizu, P. M. (2017). *pairwiseAdonis: Pairwise Multilevel Comparison using Adonis*.

Baldassarre, L., Ying, H., Reitzel, A. M., Franzenburg, S., & Fraune, S. (2022). Microbiota mediated plasticity promotes thermal adaptation in the sea anemone *Nematostella vectensis*. *Nature Communications*, *13*(1), 3804. <https://doi.org/10.1038/s41467-022-31350-z>

Becheler, R., Haverbeck, D., Clerc, C., Montecinos, G., Valero, M., Mansilla, A., & Faugeron, S.

(2022). Variation in Thermal Tolerance of the Giant Kelp’s Gametophytes: Suitability of

Habitat, Population Quality or Local Adaptation? *Frontiers in Marine Science, 9*.

https://doi.org/10.3389/fmars.2022.802535

Bengtsson, M., Sjøtun, K., Storesund, J., & Øvreås, J. (2011). Utilization of kelp-derived carbon

sources by kelp surface-associated bacteria. *Aquatic Microbial Ecology, 62*(2), 191–199.

https://doi.org/10.3354/ame01477

Bindoff, N.L., W.W.L. Cheung, J.G. Kairo, J. Arístegui, V.A. Guinder, R. Hallberg, N. Hilmi, N. Jiao, M.S. Karim, L. Levin, S. O’Donoghue, S.R. Purca Cuicapusa, B. Rinkevich, T. Suga, A. Tagliabue, and P. Williamson (2019). Changing Ocean, Marine Ecosystems, and Dependent Communities. In: IPCC Special Report on the Ocean and Cryosphere in a Changing Climate [H.-O. Pörtner, D.C. Roberts, V. Masson-Delmotte, P. Zhai, M. Tignor, E. Poloczanska, K. Mintenbeck, A. Alegría, M. Nicolai, A. Okem, J. Petzold, B. Rama, N.M. Weyer (eds.)]. Cambridge University Press, Cambridge, UK and New York, NY, USA, pp. 447-587. https://doi.org/10.1017/9781009157964.007.

Bollen, M., Pilditch, C. A., Battershill, C. N., & Bischof, K. (2016). Salinity and temperature tolerance of the invasive alga *Undaria pinnatifida* and native New Zealand kelp: Implications for competition. *Marine Biology*, *163*(9), 194. https://doi.org/10.1007/s00227-016-2954-3

Brand, T. van den. (2022). *ggh4x: Hacks for “ggplot2.”* https://CRAN.R-project.org/package=ggh4x

Caceres, M. D., & Legendre, P. (2009). Associations between species and groups of sites: Indices and statistical inference. *Ecology*, *90*(12), 3566-3574. https://doi.org/10.1890/08-1823.1

Callahan, B. J., McMurdie, P. J., Rosen, M. J., Han, A. W., Johnson, A. J. A., & Holmes, S. P. (2016). DADA2: High-resolution sample inference from Illumina amplicon data. *Nature Methods*, *13*(7), 581-583. https://doi.org/10.1038/nmeth.3869

Chen, M. Y., & Parfrey, L. W. (2018). Incubation with macroalgae induces large shifts in water column microbiota, but minor changes to the epibiota of co‐occurring macroalgae. *Molecular Ecology*, *27*(8), 1966–1979. https://doi.org/10.1111/mec.14548

Christie, H., Andersen, G. S., Bekkby, T., Fagerli, C. W., Gitmark, J. K., Gundersen, H., & Rinde, E. (2019). Shifts Between Sugar Kelp and Turf Algae in Norway: Regime Shifts or Fluctuations Between Different Opportunistic Seaweed Species? *Frontiers in Marine Science*, *6*, 72. https://doi.org/10.3389/fmars.2019.00072

Davis, K. (2022). *Factors structuring microbial communities on marine foundation species* [University of British Columbia]. https://doi.org/10.14288/1.0413811

Davis, T. R., Larkin, M. F., Forbes, A., Veenhof, R. J., Scott, A., & Coleman, M. A. (2022). Extreme flooding and reduced salinity causes mass mortality of nearshore kelp forests. *Estuarine, Coastal and Shelf Science*, *275*, 107960. https://doi.org/10.1016/j.ecss.2022.107960

Davison, I. R., & Pearson, G. A. (1996). Stress Tolerance in Intertidal Seaweeds. *Journal of Phycology*, *32*(2), 197–211. https://doi.org/10.1111/j.0022-3646.1996.00197.x

Dittami, S. M., Duboscq-Bidot, L., Perennou, M., Gobet, A., Corre, E., Boyen, C., & Tonon, T. (2016). Host—microbe interactions as a driver of acclimation to salinity gradients in brown algal cultures. *The ISME Journal*, *10*(1), 51–63. https://doi.org/10.1038/ismej.2015.104

Egan, S., Harder, T., Burke, C., Steinberg, P., Kjelleberg, S., & Thomas, T. (2013). The seaweed holobiont: Understanding seaweed—bacteria interactions. *FEMS Microbiology Reviews*, *37*(3), 462–476. https://doi.org/10.1111/1574-6976.12011

Eger, A. M., Marzinelli, E. M., Beas-Luna, R., Blain, C. O., Blamey, L. K., Byrnes, J. E. K., Carnell, P. E., Choi, C. G., Hessing-Lewis, M., Kim, K. Y., Kumagai, N. H., Lorda, J., Moore, P., Nakamura, Y., Pérez-Matus, A., Pontier, O., Smale, D., Steinberg, P. D., & Vergés, A. (2023). The value of ecosystem services in global marine kelp forests. *Nature Communications*, *14*(1), 1894. https://doi.org/10.1038/s41467-023-37385-0

Eppley, R. W., & Bovell, C. R. (1958). Sulfuric Acid in *Desmarestia*. *Biological Bulletin*, *115*(1), 101–106. https://doi.org/10.2307/1539096

Farrugia Drakard, V. Hollarsmith, J. A., & Stekoll, M. S. (2025). Hyposaline conditions impact

the early life-stages of commercially important high-latitude kelp species. *Journal of*

*Phycology*, *00*, 1-13. https://doi.org/10.1111/jpy.70003

Filbee-Dexter, K., Wernberg, T., Fredriksen, S., Norderhaug, K. M., & Pedersen, M. F. (2019). Arctic kelp forests: Diversity, resilience and future. *Global and Planetary Change*, *172*, 1–14. https://doi.org/10.1016/j.gloplacha.2018.09.005

Fox, J., & Weisberg, S. (2019). *An R Companion to Applied Regression* (Third). Sage. https://socialsciences.mcmaster.ca/jfox/Books/Companion/

Gagolewski, M. (2022). stringi: Fast and portable character string processing in R. *Journal of Statistical Software*, *103*(2), 1–59. https://doi.org/10.18637/jss.v103.i02

Gerard, V. A., DuBois, K., & Greene, R. (1987). Growth responses of two *Laminaria saccharina* populations to environmental variation. *Hydrobiologia*, *151*(1), 229–232. https://doi.org/10.1007/BF00046134

Ghaderiardakani, F., Quartino, M. L., & Wichard, T. (2020). Microbiome-Dependent Adaptation of Seaweeds Under Environmental Stresses: A Perspective. *Frontiers in Marine Science*, *7*, 575228. https://doi.org/10.3389/fmars.2020.575228

Goldsmit, J., Schlegel, R. W., Filbee-Dexter, K., MacGregor, K. A., Johnson, L. E., Mundy, C. J., Savoie, A. M., McKindsey, C. W., Howland, K. L., & Archambault, P. (2021). Kelp in the Eastern Canadian Arctic: Current and Future Predictions of Habitat Suitability and Cover. *Frontiers in Marine Science*, *18*, 742209. https://doi.org/10.3389/fmars.2021.742209

Harley, C. D. G., Anderson, K. M., Demes, K. W., Jorve, J. P., Kordas, R. L., Coyle, T. A., & Graham, M. H. (2012). Effects of climate change on seaweed communities. *Journal of Phycology*, *48*(5), 1064–1078. https://doi.org/10.1111/j.1529-8817.2012.01224.x

Hsieh, T. C., Ma, K. H., & Chao, A. (2022). *iNEXT: iNterpolation and EXTrapolation for species diversity* (3.0.0) [Computer software]. http://chao.stat.nthu.edu.tw/wordpress/software-download/

Karsten, U. (2007). Research note: Salinity tolerance of Arctic kelp from Spitsbergen. *Phycological Research*, *55*(4), 257–262. https://doi.org/10.1111/j.1440-1835.2007.00468.x

Kassambara, A. (2022a). *ggpubr: “ggplot2” Based Publication Ready Plots*. https://CRAN.R-project.org/package=ggpubr

Kassambara, A. (2022b). *rstatix: Pipe-Friendly Framework for Basic Statistical Tests*. <https://CRAN.R-project.org/package=rstatix> [Computer software].

King, N. G., Moore, P. J., Thorpe, J. M., & Smale, D. A. (2023). Consistency and Variation in the Kelp Microbiota: Patterns of Bacterial Community Structure Across Spatial Scales. *Microbial Ecology,* 85, 1265-1275. <https://doi.org/10.1007/s00248-022-02038-0>

Kraft, J. B. N., Adler, P. B., Godoy, O., James, E., Fuller, S., Levine, J. M. (2014). Community

assembly, coexistence and the environmental filtering metaphor. Community

*Phylogenetics and Ecosystem Functioning, 29(5)*, 592-599. <https://doi.org/10.1111/1365->

2435.12345.

Kumar, S., Bhavya, P. S., Ramesh, R., Gupta, G. V. M., Chiriboga, F., Singh, A., Karunasagar, I., Rai, A., Rehnstam-Holm, A.-S., Edler, L., & Godhe, A. (2018). Nitrogen uptake potential under different temperature-salinity conditions: Implications for nitrogen cycling under climate change scenarios. *Marine Environmental Research*, *141*, 196–204. https://doi.org/10.1016/j.marenvres.2018.09.001

Lahti, L., & Shetty, S. (2012). *Microbiome R package*. [Computer software].

Langdon, C., & Atkinson, M. J. (2005). Effect of elevated pCO2 on photosynthesis and calcification of corals and interactions with seasonal change in temperature/irradiance and nutrient enrichment. *Journal of Geophysical Research: Oceans*, *110*(C9). <https://doi.org/10.1029/2004JC002576>

Lavecchia, A., Fosso, B., Engelen, A. H., Borin, S., Manzari, C., Picardi, E., Pesole, G., &

Placido, A. (2024). Macroalgal microbiomes unveil a valuable genetic resource for

halogen metabolism. Microbiome, *12(1)*, 47. <https://doi.org/10.1186/s40168-023-01740->

6

Lemay, M. A., Davis, K. M., Martone, P. T., & Parfrey, L. W. (2021). Kelp‐associated Microbiota are Structured by Host Anatomy. *Journal of Phycology*, *57*(4), 1119–1130. https://doi.org/10.1111/jpy.13169

Lesser, M. P., Fiore, C., Slattery, M., & Zaneveld, J. (2016). Climate change stressors destabilize the microbiome of the Caribbean barrel sponge, *Xestospongia muta*. *Journal of Experimental Marine Biology and Ecology*, *475*, 11–18. https://doi.org/10.1016/j.jembe.2015.11.004

Li, J., Bates, K. A., Hoang, K. L., Hector, T. E., Knowles, S. C. L., & King, K. C. (2022). Experimental temperatures shape host microbiome diversity and composition. *Global Change Biology*, *29*(1), 41–56. <https://doi.org/10.1111/gcb.16429>

Lind, A. C., & Konar, B. (2017). Effects of abiotic stressors on kelp early life-history stages. *ALGAE*, *32*(3), 223–233. https://doi.org/10.4490/algae.2017.32.8.7

Lozupone, C. A., & Knight, R. (2007). Global patterns in bacterial diversity. *Proceedings of the National Academy of Sciences*, *104*(27), 11436–11440. https://doi.org/10.1073/pnas.0611525104

Madkaiker, K., Valsala, V., Sreeush, M. G., Mallissery, A., Chakraborty, K., & Deshpande, A. (2023). Understanding the Seasonality, Trends, and Controlling Factors of Indian Ocean Acidification Over Distinctive Bio‐Provinces. *Journal of Geophysical Research: Biogeosciences*, *128*(1), e2022JG006926. https://doi.org/10.1029/2022JG006926

Mansilla, A., Rosenfeld, S., Rendoll, J., Murcia, S., Werlinger, C., Yokoya, N. S., & Terrados, J. (2014). Tolerance response of *Lessonia flavicans* from the sub-Antarctic ecoregion of Magallanes under controlled environmental conditions. *Journal of Applied Phycology*, *26*(5), 1971–1977. https://doi.org/10.1007/s10811-014-0294-6

Marshall, K., Joint, I., Callow, M. E., & Callow, J. A. (2006). Effect of Marine Bacterial Isolates on the Growth and Morphology of Axenic Plantlets of the Green Alga Ulva linza. *Microbial Ecology*, *52*(2), 302–310. https://doi.org/10.1007/s00248-006-9060-x

McDevitt-Irwin, J. M., Baum, J. K., Garren, M., & Vega Thurber, R. L. (2017). Responses of Coral-Associated Bacterial Communities to Local and Global Stressors. *Frontiers in Marine Science*, *4*, 262. https://doi.org/10.3389/fmars.2017.00262

McLaren, M. R. (2020). *Silva SSU taxonomic training data formatted for DADA2 (Silva version 138)* [Dataset]. Zenodo. https://doi.org/10.5281/zenodo.3986799

McLaren, M. R., & Callahan, B. J. (2021). *Silva 138.1 prokaryotic SSU taxonomic training data formatted for DADA2* [Dataset]. Zenodo. https://doi.org/10.5281/zenodo.4587955

McMurdie, P. J., & Holmes, S. (2013). phyloseq: An R package for reproducible interactive analysis and graphics of microbiome census data. *PLoS ONE*, *8*(4), e61217.

Mikryukov, V. (2022). *metagMisc: Miscellaneous functions for metagenomic analysis* (0.0.4) [Computer software].

Monteiro, C. M., Li, H., Bischof, K., Bartsch, I., Valentin, K. U., Corre, E., Collén, J., Harms, L., Glöckner, G., & Heinrich, S. (2019). Is geographical variation driving the transcriptomic responses to multiple stressors in the kelp *Saccharina latissima*? *BMC Plant Biology*, *19*(1), 513. https://doi.org/10.1186/s12870-019-2124-0

Oksanen, J., Simpson, G. L., Blanchet, F. G., Kindt, R., Legendre, P., Minchin, P. R., O’Hara, R. B., Solymos, P., Stevens, M. H. H., Szoecs, E., Wagner, H., Barbour, M., Bedward, M., Bolker, B., Borcard, D., Carvalho, G., Chirico, M., Caceres, M. D., Durand, S., … Weedon, J. (2022). *vegan: Community Ecology Package*. https://CRAN.R-project.org/package=vegan

Parada, A. E., Needham, D. M., & Fuhrman, J. A. (2016). Every base matters: Assessing small subunit rRNA primers for marine microbiomes with mock communities, time series and global field samples. *Environmental Microbiology*, *18*(5), 1403–1414. https://doi.org/10.1111/1462-2920.13023

Peteiro, C., & Sánchez, N. (2012). Comparing salinity tolerance in early stages of the sporophytes of a non-indigenous kelp (*Undaria pinnatifida*) and a native kelp (*Saccharina latissima*). *Russian Journal of Marine Biology*, *38*(2), 197–200. https://doi.org/10.1134/S1063074012020095

Pita, L., Rix, L., Slaby, B. M., Franke, A., & Hentschel, U. (2018). The sponge holobiont in a changing ocean: From microbes to ecosystems. *Microbiome*, *6*(1), 46. https://doi.org/10.1186/s40168-018-0428-1

Posit team. (2022). *RStudio: Integrated Development Environment for R* (2022.12.0+353) [Computer software]. Posit Software. http://www.posit.co/

Provasoli, L., & Pintner, I. J. (1980). Bacteria Induced Polymorphism in an Axenic Laboratory Strain of *Ulva Lactuca* (Chlorophyceae). *Journal of Phycology*, *16*(2), 196–201. https://doi.org/10.1111/j.1529-8817.1980.tb03019.x

Qiu, Z., Coleman, M. A., Provost, E., Campbell, A. H., Kelaher, B. P., Dalton, S. J., Thomas, T., Steinberg, P. D., & Marzinelli, E. M. (2019). Future climate change is predicted to affect the microbiome and condition of habitat-forming kelp. *Proceedings of the Royal Society B: Biological Sciences*, *286*(1896), 20181887. https://doi.org/10.1098/rspb.2018.1887

Quince, C., Lanzen, A., Davenport, R. J., & Turnbaugh, P. J. (2011). Removing Noise From Pyrosequenced Amplicons. *BMC Bioinformatics*, *12*(1), Article 1. https://doi.org/10.1186/1471-2105-12-38

R Core Team. (2022). *R: A language and environment for statistical computing* [Computer software]. R Foundation for Statistical Computing. <https://www.R-project.org/>.

Rapaport M (ed)., *Salish Archipelago: Environment and Society in the Islands Within and*

*Adjacent to the Salish Sea*, 1st ed. 2024. ANU Press.

Ryan, S. A., Wohlgeschaffen, G. D., Jahan, N., Niu, H., Ortmann, A. C., Brown, T. N., King, T. L., & Clyburne, J. (2019). *State of knowledge on fate and behaviour of ship-source petroleum product spills*. Fisheries and Oceans Canada = Pêches et océans Canada.

Saha, M., & Weinberger, F. (2019). Microbial “gardening” by a seaweed holobiont: Surface

metabolites attract protective and deter pathogenic epibacterial settlement. *Journal of*

*Ecology, 107(5),* 2255–2265. https://doi.org/10.1111/1365-2745.13193

Saha, M., Ferguson, R. M. W., Dove, S., Künzel, S., Meichssner, R., Neulinger, S. C., Petersen, F. O., & Weinberger, F. (2020). Salinity and Time Can Alter Epibacterial Communities of an Invasive Seaweed. *Frontiers in Microbiology*, *10*. <https://www.frontiersin.org/articles/10.3389/fmicb.2019.02870>

Sandbakken, I. S., Sæther, M., Funderud, J., & Aasen, I. M. (2018). Acid preservation of

*Saccharina latissima* for application as a carbon source for fermentation to biofuels and

chemicals. *Journal of Applied Phycology*, *30*(6), 3581–3588.

https://doi.org/10.1007/s10811-018-1489-z

Santoro, E. P., Borges, R. M., Espinoza, J. L., Freire, M., Messias, C. S. M. A., Villela, H. D. M., Pereira, L. M., Vilela, C. L. S., Rosado, J. G., Cardoso, P. M., Rosado, P. M., Assis, J. M., Duarte, G. A. S., Perna, G., Rosado, A. S., Macrae, A., Dupont, C. L., Nelson, K. E., Sweet, M. J., … Peixoto, R. S. (2021). Coral microbiome manipulation elicits metabolic and genetic restructuring to mitigate heat stress and evade mortality. *Science Advances*, *7*(33), eabg3088. <https://doi.org/10.1126/sciadv.abg3088>

Simonson, E. J., Scheibling, R. E., & Metaxas, A. (2015). Kelp in hot water: Warming

seawater temperature induces weakening and loss of kelp tissue. Marine Ecology

*Progress Series, 537*, 89–104. https://doi.org/10.3354/meps11438

Sogn Andersen, G., Steen, H., Christie, H., Fredriksen, S., & Moy, F. E. (2011). Seasonal Patterns of Sporophyte Growth, Fertility, Fouling, and Mortality of *Saccharina latissima* in Skagerrak, Norway: Implications for Forest Recovery. *Journal of Marine Biology*, *2011*, 1–8. <https://doi.org/10.1155/2011/690375>

Smith, K. E., Aubin, M., Burrows, M. T., Filbee-Dexter, K., Hobday, A. J., Holbrook, N. J., King, N. G., Moore, P. J., Sen Gupta, A., Thomsen, M., Wernberg, T., Wilson, E., & Smale, D. A. (2024). Global impacts of marine heatwaves on coastal foundation species. *Nature Communications*, *15*(1), 5052. <https://doi.org/10.1038/s41467-024-49307-9>

Starko, S., Neufeld, C. J., Gendall, L., Timmer, B., Campbell, L., Yakimishyn, J., Druehl, L.,

Baum, J. K. (2022). Microclimate predicts kelp forest extinction in the face of direct and

indirect marine heatwave effects. *Ecological Applications, 32*(7), e2673.

https://doi.org/10.1002/eap.2673

Steneck, R. S., Graham, M. H., Bourque, B. J., Corbett, D., Erlandson, J. M., Estes, J. A., & Tegner, M. J. (2002). Kelp forest ecosystems: Biodiversity, stability, resilience and future. *Environmental Conservation*, *29*(4), 436–459. https://doi.org/10.1017/S0376892902000322

Stratil, S. B., Neulinger, S. C., Knecht, H., Friedrichs, A. K., & Wahl, M. (2014). Salinity affects compositional traits of epibacterial communities on the brown macroalga *Fucus vesiculosus*. *FEMS Microbiology Ecology*, *88*(2), 272–279. https://doi.org/10.1111/1574-6941.12292

Trevathan-Tackett, S. M., Sherman, C. D. H., Huggett, M. J., Campbell, A. H., Laverock, B., Hurtado-McCormick, V., Seymour, J. R., Firl, A., Messer, L. F., Ainsworth, T. D., Negandhi, K. L., Daffonchio, D., Egan, S., Engelen, A. H., Fusi, M., Thomas, T., Vann, L., Hernandez-Agreda, A., Gan, H. M., … Macreadie, P. I. (2019). A horizon scan of priorities for coastal marine microbiome research. *Nature Ecology & Evolution*, *3*(11), 1509–1520. <https://doi.org/10.1038/s41559-019-0999-7>

Tymon, T. M., Miller, E. P., Gonzales, J. L., Raab, A., Küpper, F. C., & Carrano, C. J. (2017).

Some aspects of the iodine metabolism of the giant kelp *Macrocystis pyrifera*

(Phaeophyceae). *Journal of Inorganic Biochemistry, 177*, 82–88.

https://doi.org/10.1016/j.jinorgbio.2017.09.003

Vadillo Gonzalez, S., Hurd, C. L., Britton, D., Bennett, E., Steinberg, P. D., & Marzinelli, E. M. (2024). Effects of temperature and microbial disruption on juvenile kelp *Ecklonia radiata* and its associated bacterial community. *Frontiers in Marine Science*, *10*. https://doi.org/10.3389/fmars.2023.1332501

van der Loos, L. M., D’hondt, S., Engelen, A. H., Pavia, H., Toth, G. B., Willems, A., Weinberger, F., De Clerck, O., & Steinhagen, S. (2023). Salinity and host drive *Ulva* ‐associated bacterial communities across the Atlantic—Baltic Sea gradient. *Molecular Ecology*, mec.16462. https://doi.org/10.1111/mec.16462

van Ginneken, V. (2018). Some Mechanism Seaweeds Employ to Cope with Salinity Stress in the Harsh Euhaline Oceanic Environment. *American Journal of Plant Sciences*, *09*(06), 1191–1211. https://doi.org/10.4236/ajps.2018.96089

Weigel, B. L., & Pfister, C. A. (2019). Successional Dynamics and Seascape-Level Patterns of Microbial Communities on the Canopy-Forming Kelp *Nereocystis luetkeana* and *Macrocystis pyrifera*. *Frontiers in Microbiology*, *10*. <https://www.frontiersin.org/articles/10.3389/fmicb.2019.00346>

Weigel, B. L., Miranda, K. K., Fogarty, E. C., Watson, A. R., & Pfister, C. A. (2022). Functional

Insights into the Kelp Microbiome from Metagenome-Assembled Genomes. *mSystems,*

*7*(3), e01422-21. <https://doi.org/10.1128/msystems.01422-21>

Weigel, B. L., Small, S. L., Berry, H. D., & Dethier, M. N. (2023). Effects of temperature and

nutrients on microscopic stages of the bull kelp (*Nereocystis luetkeana*, Phaeophyceae).

*Journal of Phycology, 59*(5), 893–907. https://doi.org/10.1111/jpy.13366

Weinbauer, M., & Rassoulzadegan, F. (2007). Extinction of microbes: evidence and potential consequences. *Endangered Species Research*, *3*, 205–215. https://doi.org/10.3354/esr003205

Wickham, H. (2016). *ggplot2: Elegant Graphics for Data Analysis*. Springer-Verlag New York. https://ggplot2.tidyverse.org

Zaneveld, J. R., McMinds, R., & Vega Thurber, R. (2017). Stress and stability: Applying the Anna Karenina principle to animal microbiomes. *Nature Microbiology*, *2*(9), Article 9. <https://doi.org/10.1038/nmicrobiol.2017.121>

Zhang, L., Cui, C., Li, X., Zhang, Z., Luo, S., Liang, G., Liu, Y., & Yang, G. (2013). Effect of

temperature on the development of *Saccharina japonica* gametophytes. Journal of

Applied Phycology, 25(1), 261–267. https://doi.org/10.1007/s10811-012-9860-y

Ziegler, M., Seneca, F. O., Yum, L. K., Palumbi, S. R., & Voolstra, C. R. (2017). Bacterial community dynamics are linked to patterns of coral heat tolerance. *Nature Communications*, *8*(1), Article 14213. https://doi.org/10.1038/ncomms14213

# Figure and Table Legends

## Main text

**Figure 1**. A) Map of the sampling sites with black arrows representing the path of main fresh water sources in the area. B) Table showing if a site was assigned as high (black) or low salinity (grey) for a field sampling round. The sampling rounds are numbered by the Julian day of the first of the two sequential field days. White cells indicate that the site was not sampled. C) Conductivity (salinity) and D) water temperature profiles across sampling years with error bars showing +/-one standard deviation around the mean. E) Representative schematic of 2022 lab experiment. Note, in D, missing temperature data from 2022 are from days where a refractometer, rather than the YSI were used to measure water parameters. We include Julian day 206 in panels B, C, and D even though we exclude this sampling round in our field data analysis because experimental trial 8 includes *Saccharina* collected during this sampling round. Also note, in E, the n = 1 for *Saccharina* bacterial samples because all individuals in the same aquaria are swabbed with the same cotton swab. Plots showing the correlation between salinity, temperature, and Julian day are in Figure S2.

**Figure 2.** *Saccharina* stress phenotypes by lab salinity treatment. A) The presence (orange) or absence (grey) of blistering by lab experimental day, experimental trial, and salinity treatment (in psu) for each *Saccharina* in lab. Experimental trial 1 lasted six days and the *Saccharina* in the 10 psu condition became green and fell apart to the touch (indicated in green). Experimental trial 2 lasted three days. Fisher’s Exact Test (p <0.001) followed by a Pairwise Fisher Test of the Day 4 data (experimental trial 3 to 8), show that the 10 psu salinity has significantly more blistering than the 20 psu and full-strength salinity on Day 4 (p ≤ 0.036). B) The ratio of dry weight to wet weight on lab Day 4. C) The ratio of wet-weight on lab Day 4 to wet-weight on Day 0. D) The estimated effective quantum yield on lab Day 4 by salinity (in psu). For B, C, and D, ANOVA output comparing the different salinity treatments are indicated in the corresponding panel. Note, trial 1 and 2 have no Day 4 observations for all panels. In addition, in panel B only, there are no Day 4 observations for experimental trial 5 because we extended the experimental time to see if the *Saccharina* would turn green. After 14 days, green *Saccharina* was not observed in any salinity treatment.

**Figure 3.** The prevalence of core ASVs on *Saccharina* in the field or in the lab. Field sample are divided on the x-axis into samples where the salinity was 20 psu or over (Field high) and where the salinity was below 20 psu (Field low). Lab samples are from Day 4 and are divided by salinity treatment. The y-axis indicates the core ASV. Bolded ASVs indicate that the ASV is core both in high and low salinity field subsets (Table S4). The fill color indicates the prevalence the ASV across all samples in the same group (x-axis).

**Figure 4.** Relative abundance of high salinity core ASVs (Table S4) in the field (A, B) and in the lab on Day 4 (C) on *Saccharina*. Plots A and B show the same samples but arranged along the salinity (A) and temperature (B) gradients observed in the field across both years. The colour gradients in panels A and B show the abiotic gradient not plotted on the x-axis. Point and line shape show the extraction kit used to extract the samples. The box between plots A and B shows the corresponding output of the linear regression. In C, the ANOVA and Tukey post-hoc comparison p-values for the ANOVA are shown. Note, all three x-axes are arranged from least to most stressful abiotic condition.

**Figure 5.** The Shannon-Wiener diversity index for the field (A,B,C) and lab Day 4 (D,E,F) samples along with the Bray-Curtis dissimilarity index of the field (G,H,I) and lab Day 4 (J,K,L) samples. Beta diversity is calculated within the same substrate type either by comparing samples within the same sampling site visit (field) or a salinity treatment (lab). The same sample types are arranged in columns and indicated in the panels. For the field samples, the point colour represents salinity and the shape (point and line) indicates the extraction kit. For all panels, the full statistical output is in Table S5, but we indicate the significant factors in the nested linear regression model (field) or Tukey post-hoc test if the ANOVA showed significant differences between groups (lab). Note, all axes are ordered from least stressful to most stressful, with the field sampling plotted along the temperature gradient while the lab samples are plotted by salinity treatment.

**Table 1.** Output of marginal PERMANOVA on field data showing the unique explanatory power of salinity and water temperature by sample type within the same extraction kit. Statistically significant results in bold. Corresponding NMDS plots are in Figure S3.

**Table 2.** Output of PERMANOVA on Day 4 lab experiment testing the explanatory power salinity treatment and experimental trial for each sample type. We ran pairwise adonis tests for salinity treatment when the main PERMANOVA was significant. *Saccharina* and water output are from a two-factor crossed PERMANOVA, while the substrate output is single factor a PERMANOVA with only salinity due to lower sample size. Statistically significant results in bold and all samples were extracted with the Zymo kit. Corresponding NMDS plots are in Figure S3.

## Supplement

**Figure S1.** NMDS plots showing differences by sample type (colour) and the extraction kit (shape) for A) both years of field samples and B) lab Day 4 samples. PERMANOVA outputs are in the corresponding panels.

**Figure S2**. Scatter plots with regression line showing the correlation between conductance (µS/cm; referred to as salinity in the text), temperature (°C), and Julian day for both 2021 and 2022. Results of Pearson’s correlation test indicated in the plot area. ­

**Figure S3.** NMDS plots by sample type showing the distribution of samples across the salinity (A:C,G:H) and temperature (D:F) gradients in the study. Panels A:C show field samples colored by salinity gradient and D:F show the same plots colored by temperature gradient. Panels G:I show lab Day 4 samples colored by salinity treatment. Note that sample number in the lab experiment are lower than the expected 16 per treatment group per sample type because of sequencing failures (sample numbers in Table S1). Point shapes indicate the extraction kit used. Corresponding output of PERMANOVA for the field (Table 1) and lab (Table 2) are in the main text.

**Figure S4.** The relative abundance of the 10 most abundant orders (A,C) or genera (B,D) in the field (A,B) and the relative abundance of these taxa in the lab samples (C,D). Field samples (A,B) are separated by extraction kit and salinity (facets) on the sampling day (x-axis) and the per-sample relative abundance is averaged across all samples from the same sampling day. Sample numbers are indicated in parentheses. All lab samples (C,D) are shown. Colours are consistent between the field and lab Day 4 plots. Tables include the output of models comparing the relative abundance of taxa in the field (Table S2) and lab Day 4 (Table S3).

**Table S1.** Number of field bacterial samples that were analyzed (successfully sequenced and passed filtering) per sample source, year, and sample type. Also, the number of samples analyzed for lab Day 4 across all 8 experimental trials. Note, all experimental trials included three salinity levels (10 psu, 20 psu, and full-strength), with two replicate aquaria per salinity level. There were three *Saccharina* per aquaria in all instances except experimental trial 1, where one of the 10 psu salinity aquaria had two *Saccharina.*

**Table S2.** Output of linear regression model comparing the per-sample relative abundance of each of the 10 most abundant orders and genera in the field *Saccharina* samples. Models were run individually for each taxa and were nested within the extraction kit, where trends in taxa relative abundance by salinity and temperature were assessed. P-values were adjusted for multiple comparisons with a Benjamini-Hochberg correction. Corresponding plot in Figure S4A,B.

**Table S3.** Output of ANOVA model comparing the per-sample relative abundance of each of the 10 most abundant orders and genera in the field in the Day 4 lab *Saccharina* samples. ANOVAs were run individually for each taxa and p-values were adjusted for multiple comparisons with a Benjamini-Hochberg correction. Corresponding plot in Figure S4C,D.

**Table S4.** Table showing the output of IndVal analysis for the field, lab, and King datasets. Taxonomy information for each ASV is provided in columns A to I. The sample type to which the ASV was assigned (*Saccharina*, water, substrate), the IndVal statistic, the specificity, and prevalence of the ASV for the assigned substrate are indicated in columns J to M (salinity ≥20) and N to Q (salinity <20). Columns R to U indicate the prevalence of the ASV in the lab by salinity (R to T) and in the King data (U). Column V indicates if the ASV was assigned as core in high (salinity ≥20) and/or low salinity (salinity <20) and/or core in the UK. We only include ASVs with a prevalence ≥0.5 in the high and/or low salinity field samples unless they are part of the UK core.

**Table S5.** Output of the best nested linear regression model by backwards AIC for the field or ANOVA for the lab Day 4 samples. From left to right, the columns indicate if the samples analyzed are from the field or lab, if the model is analyzing alpha (Shannon-Wiener) or beta diversity (Bray-Curtis Dissimilarity), the type of mode, the sample type analyzed, the output of the overall model, the output for each factor in the model (field data only), and the output of the Tukey post-hoc test (lab only).
